# Bioprospecting of Beneficial Bacteria Traits Associated With Tomato Root in Greenhouse Environment Reveals That Sampling Sites Impact More Than the Root Compartment

**DOI:** 10.3389/fpls.2021.637582

**Published:** 2021-04-13

**Authors:** Alice Anzalone, Mario Di Guardo, Patrizia Bella, Farideh Ghadamgahi, Giulio Dimaria, Rosario Zago, Gabriella Cirvilleri, Vittoria Catara

**Affiliations:** ^1^Department of Agriculture, Food and Environment, University of Catania, Catania, Italy; ^2^Department of Agricultural, Food and Forest Sciences, University of Palermo, Palermo, Italy; ^3^Department of Plant Protection, Faculty of Agriculture, Ferdowsi University of Mashhad, Mashhad, Iran; ^4^Agronomist, Ragusa, Italy

**Keywords:** microbiome, tomato, PGPR, BCA, endorhizosphere

## Abstract

Tomato is subject to several diseases that affect both field- and greenhouse-grown crops. To select cost-effective potential biocontrol agents, we used laboratory throughput screening to identify bacterial strains with versatile characteristics suitable for multipurpose uses. The natural diversity of tomato root–associated bacterial communities was bioprospected under a real-world environment represented by an intensive tomato cultivation area characterized by extraseasonal productions in the greenhouse. Approximately 400 tomato root–associated bacterial isolates, in majority Gram-negative bacteria, were isolated from three compartments: the soil close to the root surface (rhizosphere, R), the root surface (rhizoplane, RP), and the root interior (endorhizosphere, E). A total of 33% of the isolates produced siderophores and were able to solubilize phosphates and grow on NA with 8% NaCl. A total of 30% of the root-associated bacteria showed antagonistic activity against all the tomato pathogens tested, i.e., *Clavibacter michiganesis* pv. *michiganensis*, *Pseudomonas syringae* pv. *tomato*, *Pseudomonas corrugata* and *Xanthomonas euvesicatoria* pv. *perforans*, and *Fusarium oxysporum* f. sp. *lycopersici*. We found that the sampling site rather than the root compartment of isolation influenced bacterial composition in terms of analyzed phenotype. This was demonstrated through a diversity analysis including general characteristics and PGPR traits, as well as biocontrol activity *in vitro*. Analysis of 16S rRNA gene (rDNA) sequencing of 77 culturable endophytic bacteria that shared multiple beneficial activity revealed a predominance of bacteria in Bacillales, Enterobacteriales, and Pseudomonadales. Their *in vitro* antagonistic activity showed that *Bacillus* species were significantly more active than the isolates in the other taxonomic group. *In planta* activity against phytopathogenic bacteria of a subset of *Bacillus* and *Pseudomonas* isolates was also assessed.

## Introduction

Tomato is one of the most widely grown vegetables and represents a major agricultural industry, with a global production of more than 180 million tons in 2018^1^. It is one of the vegetables that is most consumed in the world (second to potatoes) and is also one with the most beneficial effects on human health (He et al., 2006). Plant diseases seriously impact tomatoes in several geographical areas worldwide. At least 140 viral species have been reported, some of which have emerged in greenhouse grown tomato plants (Moriones and Verdin, 2020). Several bacterial species were described causing leaf spots, vascular diseases and roots (Catara and Bella, 2020). In addition, intensive greenhouse cropping systems have greatly facilitated the development of fungal and fungal-like diseases (Bardin and Gullino, 2020).

The intensive management required to mitigate serious economic losses has encouraged the search of alternative approach for the control of tomato diseases, including the use of biological control agents (BCAs) (Singh et al., 2017).

Instead of an independent entity, according to the most recent definition, the plant is regarded as a holobiont or “super organism” that is integrated with the microorganisms associated with it (microbiota) and their genetic information (often referred to as the microbiome) (Vandenkoornhuyse et al., 2015). The microbiome is involved in multiple plant functions, ranging from nutrition to resistance to biotic and abiotic factors (Hardoim et al., 2008; Mendes et al., 2011). The productivity, vigor, and resistance of the plant is therefore not only the direct consequence of the genetic makeup of the plant itself, but also of its microbiome or set of microorganisms (Philippot et al., 2013; Berg et al., 2016).

There is a relatively large body of information on the tomato microbiome as many studies have explored the mechanisms of microorganism selection in different compartments of the plants, also identifying beneficial microorganisms and potential candidates for biological control. Metagenomic studies based on amplicon sequencing have identified the microbial communities associated with different tomato plant organs (Ottesen et al., 2013). An interesting gradient with regard to the distance of each plant part from the soil has been observed as microbial diversity decreases as the distance from the soil increases (Ottesen et al., 2013; Dong et al., 2019). The most attention has been paid to the rhizosphere where there is a highly active microbial interaction as exudates released by plant roots are the main food source for microorganisms and a driving force for their population density and activities (Raaijmakers et al., 2009; Bulgarelli et al., 2013). A subset of rhizospheric microorganisms penetrates the plant roots and colonizes the endosphere (horizontal transmission) (Compant et al., 2010). Vertical transmission of bacterial endophytes via seeds has also been reported in different crops (Truyens et al., 2015; Cavazos et al., 2018; Rezki et al., 2018). These endophytes reside within plants with no obvious negative effects on the host, contributing to their growth and development and the ability to adapt to adverse conditions (Vandenkoornhuyse et al., 2015; Sinno et al., 2020). Tomato rhizosphere and endorhizosphere microbial communities have been investigated according to soil characteristics (Poli et al., 2016), genotypes (French et al., 2020; Taffner et al., 2020), crop management (Allard et al., 2016), rootstocks (Poudel et al., 2019), and soilborne pathogen infections (Li et al., 2014; Tian et al., 2015; Larousse et al., 2017). Overall, the results suggest that the tomato endophytic microbiome is mainly horizontally transferred from the soil environment (Poli et al., 2016; Chialva et al., 2018), but also vertically transmitted via seeds from where it can be transmitted to the subsequent plant generation (Bergna et al., 2018). Culture-dependent methods have been used to study microbial communities of the tomato root environment, mainly aimed at selecting plant growth-promoting rhizobacteria (PGPRs) and biocontrol agents (Abbamondi et al., 2016; Abdeljalil et al., 2016; Tian et al., 2017; Attia et al., 2020; Saqib et al., 2020). Microorganisms may have a neutral, pathogenic, or beneficial interaction with their host plant, and together with plant pathogens, beneficial microorganisms in the plants can interact in different ways with the plant (Raaijmakers et al., 2009). The main roles of beneficial microorganisms are biostimulation (or phytostimulation), i.e., the direct promotion of plant growth by the production of phytohormones (Bloemberg and Lugtenberg, 2001); biofertilization (Bashan, 1998), i.e., the promotion of plant growth generated by the microorganisms that facilitate accessibility to essential nutrients or increase the supply of nutrients to the plant; and biocontrol activity, i.e., the ability to control plant pathogens [biological control agents (BCAs)] through the competition for space and nutrients, the production of antibiotic substances, or the induction of resistance mechanisms (Bloemberg and Lugtenberg, 2001; Heimpel and Mills, 2017). Bacteria that share at least two of these mechanisms of action are known as PGPRs (Glick, 1995). The use of microorganisms, alone or combined in consortia, is foreseen as a method to positively modify the plant microbiome in order to improve the quantity and quality of agricultural crops. It has shown great potential as a low-environmental-impact alternative to agrochemicals and fertilizers (Ciancio et al., 2016; Woo and Pepe, 2018; Compant et al., 2019).

Microbiome studies based on metagenomics have greatly contributed to the understanding of the complex network established between the tomato rhizosphere and its microbiota. However, the information gained to date mainly refers to identify the microbiota, understanding where they come from and what the main driving conditions are that modify the microbiota but not what their actual role is. To date, cultivation-dependent methods have been used to isolate and characterize bacterial isolates from tomato plants exhibiting appreciable PGPR and BCA capabilities (Enya et al., 2007; Amaresan et al., 2012; Xu et al., 2014; Abbamondi et al., 2016; Romero et al., 2016; Tian et al., 2017; Attia et al., 2020; Saqib et al., 2020).

Vacheron et al. (2016) and Besset-Manzoni et al. (2019) used a systematic sampling method for comparing bacterial populations on maize and wheat, respectively, we adopted a similar approach in the current study. A similar approach was also used by Lemanceau et al. (1995) that demonstrated that *Pseudomonas* communities of the root compartments are influenced by plant species (flax and tomato). While they faced the problem with biochemical features and genotyping aimed at taxonomic resolution of the problem (nowadays approached by metabarcoding), we focused on the phenotyping of the beneficial traits. This approach was applied within the framework of a project on tomato microbiota aimed at the selection of bacterial isolates to be used in microbial consortia for seed or plantlet bacterization in the nursery. We investigated the diversity of the cultivable bacterial population associated with the tomato root environment. In addition, we particularly focused on bacterial endophytes in terms of being beneficial biocontrol agents. Samples were collected from farms from a restricted area specialized in the intensive cultivation of tomato under a greenhouse environment in Ragusa province (Sicily). This is the principal production area in Italy that uses greenhouses covered by plastic films, with more than half of the national tomato production. This area is characterized by sandy soil, high salinity conditions, and favorable climatic conditions that permit extraseasonal productions (up to two cycles a year), above all of cherry tomato typologies.

The main findings of our work were as follows: (i) cultivable bacterial population sizes in the root are higher in the rhizosphere and in the rhizoplane than in the endosphere compartment; (ii) the site of isolation (i.e., farm and agricultural conditions) rather than the root compartment drives the phenotypic characteristic of bacterial populations; (iii) efficient cultivable bacteria from tomato endorhizosphere belong to Bacillales, Pseudomonadales, and Enterobacteriales order; (iv) *Bacillus* species are significantly more effective in inhibiting tomato plant pathogens *in vitro*; (v) preliminary *in vivo* results showed that some *Pseudomonas* and *Bacillus* isolates from endorhizosphere may protect tomato plants against plant pathogenic bacteria and thus deserve further investigation.

## Materials and Methods

### Sampling of Tomato Root–Associated Bacteria

Tomato plants (*Solanum lycopersicum* L.) were grown in unheated greenhouses on four farms located in an area devoted to greenhouse vegetable production in Ragusa province (Sicily, Italy). The positions, soil proprieties, and genotypes are shown in Table 1. Plants were grown in agricultural soil and watered by drip irrigation, following standard agronomical practices. Five healthy plants from each farm were randomly selected from the central rows of each greenhouse, and their associated root material was collected at the fruiting stage, in March 2018. Plant stems were cut 30 cm above the root collar, and the five root systems were placed in a plastic bag and immediately transferred to the laboratory in a cooler. The samples were preserved at 4°C and processed within 24 h.

**TABLE 1:** Data on sampling sites and number of bacterial isolates from the tomato root environment.

	**Farm 1**	**Farm 2**	**Farm 3**	**Farm 4**
**Position**				
Locality	Ispica (RG, Italy)	Ispica (RG, Italy)	Ragusa (Italy)	Vittoria (RG, Italy)
Geographic coordinates	36°42′35.62″ N 14°57′36.13″ E	36°42′59.08″ N 14°58′59.98″ E	36°51′3.24″ N 14°27′41.40″ E	36°56′40.49″ N 14°23′42.37″ E
**Soil properties**				
Soil texture	Sandy clay calcareous loamy	Sandy calcareous	Sandy calcareous	Sandy calcareous loamy
Organic matter (%)	1.93	2.1	1.07	2.5
Ph	7.57	7.72	7.71	7.7
Electrical conductivity (mmhos cm^–1^)	2.85	8.45	2.13	3.52
P (mg kg^–1^)	102	655	135	155
Zn (mg kg^–1^)	1.9	11.6	6.9	5.9
Mn (mg kg^–1^)	22.8	32.4	14.4	13.2
Cu (mg kg^–1^)	6.1	13.2	4.8	14.4
Fe (mg kg^–1^)	12.2	49.2	15.6	4.6
K (mg kg^–1^)	391	507	96	747
Mg (mg kg^–1^)	254	327	203	529
Na (mg kg^–1^)	158	340	156	290
Ca (mg kg^–1^)	221	925	202	290
**Tomato genotype**				
Typology	Cherry	Mini plum	Cherry	Mini plum
Genotype	Casarino F1	Dulcemiel F1	Creativo F1	Miele F1
Number of isolates	70	132	85	136

Tomato root–associated bacteria were isolated from three compartments: the soil close to the root surface (rhizosphere, R), the root surface (rhizoplane, RP), and the root interior (endorhizosphere, E). Samples were processed according to the protocol described by Normander and Prosser (2000) and Wieland et al. (2001), with some modifications, as follows:

– *Rhizosphere (R)*: Roots were shaken carefully to remove non-adhering soil. Five grams of soil adhering to the roots was manually collected and transferred in sterile 50 mL centrifuge tubes containing 20 mL of sterile saline buffer (0.85% NaCl) and then mixed thoroughly by vortex for 2 min.
– *Rhizoplane (RP)*: Roots (approximately 5 g), from which the rhizospheric soil had been dislodged, were soaked in 20 mL of sterile saline buffer (0.85% NaCl) and mixed thoroughly by vortex for 5 min.
– *Endorhizosphere (E)*: After treatment for rhizoplane bacteria extraction, roots (approximately 5 g) were sterilized with 75% ethanol (2 min), 50% sodium hypochlorite solution (2 min), and ethanol 75% (1 min) and rinsed five times in sterile distilled water (SDW). Sterility was assessed by placing the sterilized roots on potato dextrose agar (PDA, Oxoid, Milan, Italy) at 27°C for 4–7 days. A lack of bacterial growth ensured the sterility of the root surfaces. The roots were then homogenized with a sterile pestle and mortar in 20 mL of sterile saline buffer (0.85% NaCl).

### Culturable Bacterial Population Sizes

Serial 10-fold dilutions in sterile saline buffer (0.85% NaCl) were prepared from each extract (R, RP, and E), and 0.1 mL of each dilution was plated onto the following media: plate count agar (Lickson, Palermo, Italy), supplemented with cycloheximide (100 mg⋅mL^–1^) to isolate and quantify the cultivable fast-growing bacteria; King’s medium B agar, supplemented with cycloheximide (100 mg⋅mL^–1^) to count the fluorescent *pseudomonads* (King et al., 1954). In order to isolate spore-forming bacteria, each extract was heat-treated (90°C) for 10 min and mixed by vortex for 1 min (Janštová and Lukášová, 2001; Manzum and Al Mamun, 2018), and after serial 10 dilutions, 0.1 mL of suspensions were plated onto nutrient agar (NA; Oxoid, Milan, Italy) with cycloheximide (100 mg⋅mL^–1^). For each compartment, dilution, and medium, three replicates were performed. The inoculated plates were incubated at 27°C for 48–72 h. The number of bacterial colony-forming units (CFUs) was then counted by visual observation, and selected colonies were isolated in pure culture. The culturable population of tomato-associated bacteria was expressed as the log of the number of CFUs per gram of soil (rhizosphere) or of roots (rhizoplane and endorhizosphere).

The root-associated bacteria were selected from plates containing 30–300 colonies, i.e., typically 10^–2^ endorhizosphere (1:100) and 10^–5^ rhizosphere and rhizoplane dilutions (1:100,000). Bacterial colonies were selected according to their macromorphological diversity (size, color, and morphology of the colony), streaked twice on PDA medium, and checked for purity. After 24 h of incubation, single colonies of the selected isolates were picked off and individually inoculated with a sterile toothpick in 96-microwell cell culture plates (Nunc^TM^ MicroWell^TM^ 96-well, collagen type i–treated, flat-bottom microplate, Thermo Fisher Scientific) containing Luria–Bertani (LB) broth. After overnight incubation, the wells were supplemented with 15% glycerol and stored at −80°C. For routine growth, isolated bacteria were picked off from stock cell cultures using an 8 × 6 replica plater (Sigma).

### Phenotypic Characterization of Bacterial Isolates

#### General and PGPR Traits

Colonies of bacterial isolates were preliminarily characterized in terms of color, shape, opacity, size, and morphology. The Gram reaction was performed using the 3% KOH test (Schaad et al., 2001). The following features were assessed: siderophore production, salt tolerance, and phosphate solubilization. Bacterial isolates from 24 h-old cultures on PDA were plated using the replica-plate device (48 isolates per plate) in the respective media, and results were recorded for up to 3 days of incubation at 28°C. All strains were tested in three independent replicates.

To detect the phosphate solubilizing bacteria, bacterial isolates were streaked onto Pikovskaya’s agar medium (Pikovskaya, 1948). Strains that induced a clear zone around the colonies were considered as positive. Siderophore production was determined on chrome-azurol S (CAS) medium (Schwyn and Neilands, 1987). The formation of orange to yellow halos around the colonies confirmed the production of siderophores. The salt tolerance was evaluated by inoculating the isolates on three NA plates containing 0, 2, and 8% NaCl. Bacterial isolates were classified based on their growth at different NaCl concentrations in the medium.

#### Antimicrobial Activity Against Tomato Pathogens

To phenotype the biocontrol activity potential of the tomato root–associated bacteria, these bacteria were screened for their antimicrobial activity against a set of tomato plant pathogens usually occurring in the area: the Gram-positive bacterium, *Clavibacter michiganensis* subspecies *michiganensis* strain PVCT156.1.1 (*Cmm*), and the Gram-negative bacteria, *Pseudomonas corrugata* strains CFBP5454 (*Pco*), *P. syringae* pv. tomato strains PVCT28.3.1 (*Pto*), *Xanthomonas euvesicatoria* pv. *perforans* strain NCPPB4321 (*Xep*), and *Fusarium oxysporum* f. sp. *lycopersici* strain Saitama ly2 (*Fol*) (Table 2).

**TABLE 2:** Tomato pathogens, bacteria and fungi, used in this study.

**Species**	Strain*	**Origin**	**Disease**	**References**
*Pseudomonas corrugata* (*Pco*)	CFBP 5454	Italy	Pith necrosis	Trantas et al., 2015
*Pseudomonas syringae* pv. *tomato* (*Pto*)	PVCT 28.3.1	Italy	Bacterial speck	Bella and Catara, 1998
*Clavibacter michiganensis* subsp. *michiganensis* (*Cmm*)	PVCT 156.1.1	Italy	Bacterial wilt and canker	Ialacci et al., 2016
*Xanthomonas euvesicatoria* pv. *perforans* (*Xep)*	NCPPB 4321^*T*^	United States	Bacterial spot	Constantin et al., 2016
*Fusarium oxysporum* f. sp. *lycopersici* (*Fol*)	Saitama ly2	Japan	*Fusarium* wilt	Hirano and Arie, 2006

*CFBP, International Center for Microbial Resources, French Collection for Plant-associated Bacteria, INRA, Angers, France; NCPPB, National Collection of Plant Pathogenic Bacteria, Fera, York, United Kingdom; PVCT, Patologia Vegetale, University of Catania, Catania, Italy.

The antagonistic activity against plant pathogenic bacteria was tested on large PDA plates (Ø 20 cm). Bacterial suspensions in SDW (OD_600_ = 0.01) were obtained from overnight cultures of the plant pathogenic bacteria in nutrient broth. A sterile swab was dipped into the inoculum tube and used to inoculate the plates by streaking the swab three times over the entire agar surface and then rotating the plate approximately 60 degrees each time, as in the Kirby–Bauer antibiotic resistance test (Hudzicki, 2009). After drying, the plates were spot-inoculated with bacterial isolates for testing using sterile toothpicks. Forty-eight bacteria were inoculated on each plate and incubated at 28°C for 1–5 days. The antagonistic activity was expressed as the width (mm) of the growth inhibition area of phytopathogenic bacterium around the bacterial colonies. The experiments were performed in three independent replicates.

To test the antagonistic activity against *Fol*, bacterial isolates were spot inoculated near the border of small PDA plates (Ø 6 cm, four bacteria per plate). After 24 h of incubation at 28°C, a mycelial plug (0.5 × 0.5 cm) from a 4 day-old culture of *Fol* was placed in the center of each plate. Plates inoculated only with the fungal plug served as the control. All strains were tested in three independent replicates. The antifungal activity was expressed as the percentage of growth inhibition (PGI) according to Vincent (1947): PGI (%) = 100 ⋅ (GC − GT)/GC, where GC represents the mean value of the fungus radius in the absence of the bacteria (control), and GT represents the mean value of the fungus radius in the presence of antagonistic bacteria (treatment). Antagonist activity was recorded after incubation at 28°C for up to 5–7 days. The comparison of the antagonistic activity of the bacterial strains was based on two arbitrary 0–3 scales. The antibacterial activity was scored based on the growth inhibition area size as follows: 0, no antagonism; 1, < 3 mm; 2, ≥ 3 and < 10 mm; 3, > 10 mm. Antifungal activity was scored based on the PGI against *Fol* as follows: 0, no inhibition, 1, PGI < 30%; 2, PGI 30–60%; 3, PGI > 60%.

### Molecular Identification of the Bacterial Endophytes

The 16S rRNA gene region was amplified and sequenced for taxonomic identification. Bacterial DNA targets for colony PCR were prepared by thermal lysis (10 min at 100°C) of cell suspensions (OD_600_ = 0.01) in 200 μL of SDW. PCR amplicons were generated using the universal 16S rRNA primer pair, 27F (5′-AGAGTTTGATCCTGGCTCAG-3′) and 1492R (5′-GGTTACCTTGTTACGACTT-3′) primer set (Edwards et al., 1989; Lane, 1991). Master mixtures included 1 × Taq&Go G2 Hot Start colorless PCR Master Mix (Promega), 0.5 μM of each primer, and 1 μL of template in a total volume of 15 μL. Reactions were performed in a thermal cycler GeneAmp PCR system 9700, with the following thermal protocol: DNA denaturation for 5 min at 95°C, amplification (35 cycles) at 94°C for 1 min, 50°C for 1 min, and 72°C for 1 min, and ended with 10-min extension at 72°C. The 1,400 bp PCR products were analyzed by agarose gel electrophoresis [1.0% (wt/vol) agarose, 90 V, 50 min]. The DNA amplicons were quantified and sequenced by BMR Genomics (Padova, Italy).

### Sequence Analysis and Construction of a Phylogenetic Tree

The sequences were searched against the nucleotide collection database at the National Center for Biotechnology Information (NCBI) nucleotide database using Basic Local Alignment Search Tool BLASTN^2^. Taxonomy information was assigned by the NCBI Taxonomy database according to the highest score sequence. Highly homologous sequences were aligned using Clustal-W algorithm within MEGA X; the regions of ambiguous alignment were edited manually and a neighbor-joining tree was generated (Kumar et al., 2018). Sequences were aligned by Clustal W within MEGA X. A phylogenetic tree was built including 16S rRNA gene sequences of the type strains identified by BLAST.

### *In vivo* Biocontrol Activity Assays

#### Bacterial Pathogens and Antagonists’ Inoculum Preparation

Of the 77 endophytes belonging to the genera *Pseudomonas* and *Bacillus*, 10 were selected to evaluate their biocontrol activity *in vivo* on tomato plants against *Cmm* and *Xep*. The endophytes were selected on the basis of their taxonomy, i.e., representativeness of the species and the results of *in vitro* test (Supplementary Table 1). The strains selected were *Bacillus velezensis* strains 261, 263, 265, and 306 and *Bacillus megaterium* strain 268; *Pseudomonas citronellolis* strain f1, *Pseudomonas monteilii* strain f53, and *Pseudomonas plecoglossicida* strains 171, 172, and f56. The inoculum of both pathogens and putative biocontrol agents was prepared from bacterial cells grown for 48 h on NDA. Single colonies were transferred into LB broth and incubated at 27°C ± 1°C for 24 h in an orbital shaker at 150 revolutions/min (rpm). The bacterial cultures were centrifuged at 7,500 rpm for 15 min. The pellets were resuspended in sterile tap water, and the density adjusted to 2 × 10^8^ CFU⋅mL^–1^ (OD_600_ = 0.1).

#### Plant Material and Inoculation of Bacterial Endophytes

Plantlets of tomato SIR ELYAN F1 3 weeks after germination were obtained from a local nursery and transplanted into square pots (8 cm side) containing nursery peat. In each trial, the pots were arranged in a completely randomized design, with 15 replicates per treatment. Independent trials were set up to assess the effect of the 10 endophytic strains on (i) PGP activity, (ii) biocontrol of bacterial canker, and (iii) biocontrol of bacterial spot. Plants were maintained in a growth chamber at 24°C ± 2°C, 68–80% RH, with 16 h of light and 8 h of darkness daily. They were watered as required with the same amount of tap water per pot. All experiments were conducted in duplicate. In all trials, bacterial endophytes were inoculated by soil drenching with 20 mL inoculum. In the PGP trial 30 days after soil treatment, tomato seedlings were harvested. Height, fresh, and dry weight of roots and shoots and shoot-to-root ratio were measured. To determine the dry weight, the samples were dried at 105°C for 24 h. These parameters were compared to mock control plants drenched with tap water.

#### Plant Challenge With Bacterial Pathogens

Tomato seedlings were inoculated with *Cmm*, bacterial suspension 7 days after treatment with the putative BCAs or water (negative control). Aliquots of 20 mL of *Cmm* were poured into the soil near the stem crown. The roots were then damaged in order to facilitate bacterial penetration by inserting a scalpel at three points located 2 cm from the stem. Bacterial canker symptoms were recorded weekly for 1 month using a 0–5 disease scale developed for root inoculations, where 0 = no symptoms, 1 = chlorosis and loss of turgor, 2 = wilt in 1 or 2 leaves, and/or cankers < 0.5, 3 = wilt in 3 or more leaves, and/or cankers > 0.5, 4 = fully withered plants, and 5 = dead plants (Bella et al., 2012).

The area under disease progress curve (AUDPC) was calculated using weekly recorded data, as described by Madden and Campbell (1990). Using hand-trigger sprayers 3 days after the soil treatment with the putative BCAs or water (negative control), tomato seedlings were spray inoculated with *Xep* onto the abaxial and adaxial leaf surfaces of four replicate tomato plants until runoff. The inoculated plants were preincubated and postincubated for 1 day under transparent polyethylene sheets to increase the RH near 100% to promote bacterial penetration. Ten tomato leaflets per plant were sampled randomly 10 days after pathogen inoculation. Lesions on individual leaflets were counted and leaflet area determined; disease severity was quantified as number of lesions/cm^2^ leaflet area (Ji et al., 2006). The leaflet area was obtained by image processing and analysis in Java (ImageJ software). Disease severity data were log transformed and subjected to analysis of variance (ANOVA). Percentage reduction in disease severity compared to the pathogen-only control was calculated according to Ji et al. (2006).

### Statistical Analysis

The results of the screening indices were used to perform a principal component analysis (PCA) to detect patterns of similarity among the tomato root–associated bacteria. The PCA was calculated on binary data (0, isolate negative to the test; 1, isolate positive to test) using the “prcomp” function of the “stat” R package (R Core Team, 2013). PCA biplot and loading projections were visualized through the “factoextra” R package (Kassambara and Mundt, 2016). Mosaic plots were drawn using the “stat” R package; the same package was also used to compute ANOVA and the *post hoc* Tukey–Kramer test. Data of biocontrol assays were analyzed by ANOVA using STATGRAPHICS Plus 5. Mean values were compared using the Student–Newman–Keuls test.

## Results

### Bacterial Population Size in Tomato Root Environment

**FIGURE 1:**
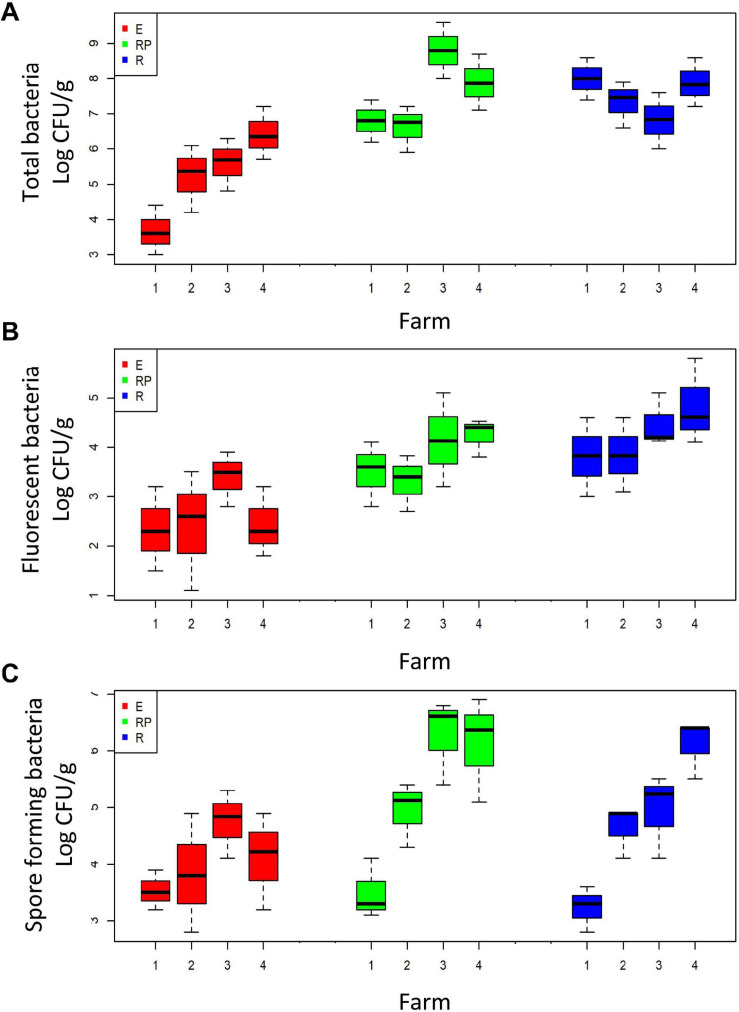
Boxplot of the total **(A)**, fluorescent **(B)**, and spore-forming **(C)** cultivable bacteria in the three root compartments: rhizosphere (R, in red), rhizoplane (RP, in green), and endorhizosphere (E, in blue). Bacteria are grouped according to the farm on which they were collected (*x*-axis).

Cultivable population sizes of total, fluorescent, and spore-forming bacteria in the rhizospheric soil (R) of the four farms ranged from 6.8 to 8.8, from 3.8 to 4.5, and from 3.3 to 6.4 log CFU ⋅ g^–1^, respectively (Figures 1A–C). On each farm, the populations were higher in the rhizosphere than in the endorhizosphere (E) (ANOVA; *p* < 0.05) (Figures 1A–C). Population sizes in the rhizoplane (RP) and in the endorhizosphere ranged from 6.8 to 8.1 and from 3.7 to 6.4 log CFU ⋅ g^–1^ for total bacteria, and from 3.8 to 4.6 and from 2.3 to 3.5 log CFU ⋅ g^–1^ for fluorescent bacteria, in the two root compartments, respectively (Figures 1A,B). The population sizes of spore-forming bacteria ranged from 3.6 to 6.4 and from 3.5 to 4.8 log CFU⋅g^–1^, for the two root compartments, respectively (Figure 1C).

### Beneficial Phenotypes of Bacteria From the Root Environment of Tomato Grown in Agricultural Soil

A total of 424 culturable bacterial strains were obtained in pure culture from the isolation plates of the four farms (70, 132, 85, and 136 isolates for farms 1–4, respectively). The percentage of Gram-negative bacteria in the three compartments was 61, 86, and 78% for R, RP, and E, respectively. Among these, fluorescent pseudomonas represented approximately 18.2, 38.6, and 43.2% of the isolates obtained from the R, RP, and E, respectively (Supplementary Table 1).

**FIGURE 2:**
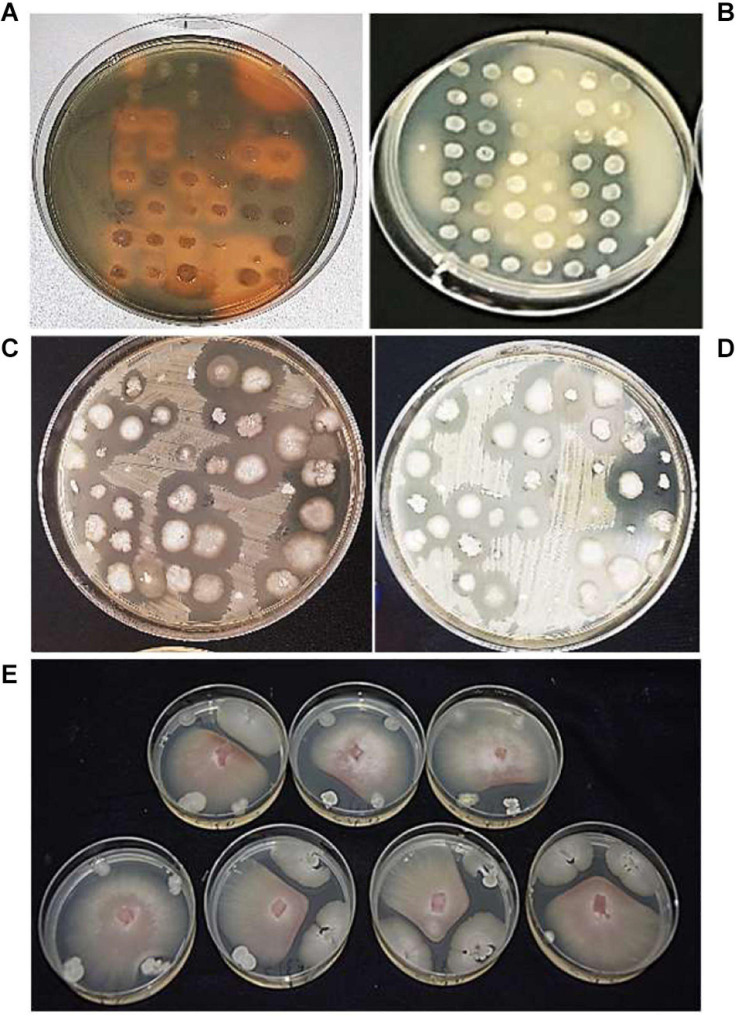
Bacterial isolates, tested for **(A)** siderophore production, orange halos indicate siderophore positive results; **(B)** phosphate solubilization, cleared halos indicate phosphate solubilization positive results; antagonistic activity against **(C)** *Xanthomonas euvesicatoria* pv. *perforans* (*Xep*), **(D)** *Pseudomonas syringae* pv. tomato (*Pto*), and **(E)** *Fusarium oxysporum* f. sp. *lycopersici* (*Fol*).

A total of 83.5, 86, and 89% of bacterial isolates from the R, RP, and E, respectively, were able to grow in up to 8% NaCl (Supplementary Table 1). The production of siderophores on CAS agar was found in a similar relative frequency in the three rhizoplanes (33, 34, and 30% in R, RP, and E) (Figure 2A and Supplementary Table 1). A total of 64% of the endophytic isolates showed an ability to solubilize insoluble organic phosphate, whereas the number of isolates showing the same characteristic was 46.5 and 29.5% in R and RP, respectively (Figure 2B and Supplementary Table 1). All the isolates exhibited at least one of the three PGP traits tested (siderophore production, phosphate solubilization, and tolerance to salinity), and most of the strains tested positive for at least two of the three traits, with 139 of the 424 isolates tested showing the three positive features: tolerance to salinity, siderophore production, and phosphate solubilization (Supplementary Table 1).

**FIGURE 3:**
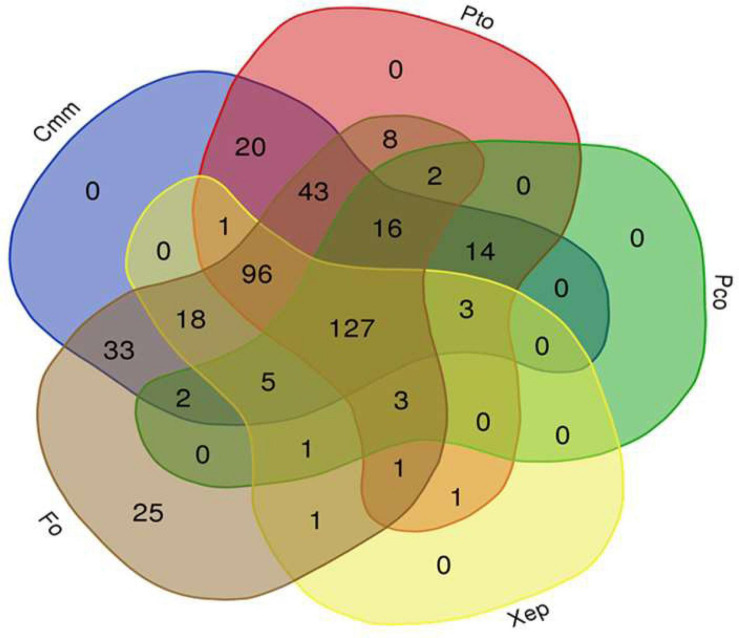
Venn diagram showing the antagonist activity of a collection of 424 bacterial isolates obtained from tomato root environment against the tomato phytopathogenic bacteria *Clavibacter michiganensis* subsp. *michiganensis* (*Cmm*), *Pseudomonas corrugata* (*Pco*), *P. syringae* pv. *tomato* (*Pto*), and *Xanthomonas euvesicatoria* pv. *perforans* (*Xep*), and the fungus *Fusarium oxysporum* f. sp. *lycopersici* (*Fol*) (http://bioinformatics.psb.ugent.be/webtools/Venn/).

The tomato root–associated bacterial collection was further screened for the antagonistic ability to inhibit *in vitro* the growth five detrimental tomato phytopathogens (Table 2 and Figures 2C–E). All isolates were therefore tested against the Gram-positive *C. michiganensis* subspecies *michiganensis* strain PVCT156.1.1 (*Cmm*), *P. corrugata* strain CFBP5454 (*Pco*), *Pseudomonas syringae* pv. tomato strain PVTC28.3.1 (*Pto*), *X. euvesicatoria* pv. *perforans* strain NCPPB4321 (*Xep*), and the fungus *F. oxysporum* f. sp. *lycopersici* strain Saitama ly2 (*Fol*). Approximately 30% of the tomato root–associated bacteria (127 of 424 isolates) showed antagonistic activity against all the tested bacterial phytopathogens and *Fol* (Figure 3 and Supplementary Table 1). Of these, 42, 26, and 31% were isolated from R, RP, and E compartments, respectively. The highest activity in terms of the number of antagonistic strains but also effectiveness in terms of inhibition zone was observed against *Cmm* (88% of the isolates) (Supplementary Figure 1). Among this group, 98% were ranked within class 3 (inhibition halo > 10 mm). The lowest number of antagonistic bacteria was detected against *Pco* (40%), and the antagonistic activity was ranked with 1 in the scale of activity (< 3 mm). An intermediate behavior was observed against the other two plant pathogenic bacteria (Supplementary Figure 1).

The *in vitro* inhibition of Fol was observed, although to different extents, by all but three tomato root–associated bacterial isolates (Supplementary Figure 1). The percentage of bacterial isolates with antifungal activity was the highest for RP (33%), followed by R (32%) and E (21%). PGI values of the fungal colonies ranged from 8 to 100% after incubation for 6 days at 24°C (when the colonies on control plates reached the margin). Based on growth inhibition scores (0–3) exhibited toward *Fol*, 214 isolates were ranked in class 2, indicating that their relative percentages of growth inhibition were less than 30% (Supplementary Figure 1). Interestingly, 60 isolates led to more than 60% inhibition of pathogen growth and were thus ranked in class 3 (Supplementary Figure 1).

### Source of Isolation Drives Beneficial Traits of Bacterial Isolates

**FIGURE 4:**
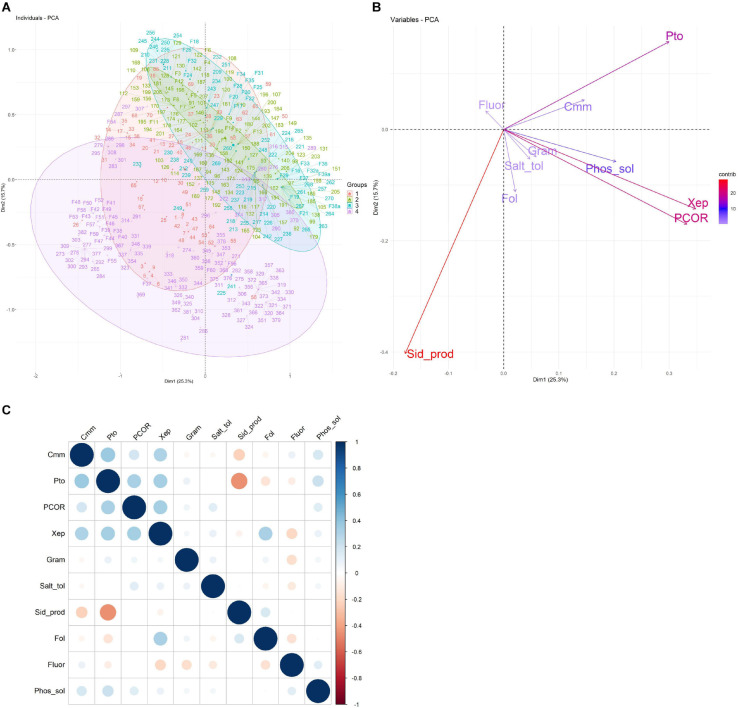
Principal component analysis (PCA) of the characteristics related to the collection of 424 bacterial isolates. **(A)** The first two principal components are shown in a biplot, and bacterial isolates are colored according to the farm on which they were isolated. **(B)** Loading plot of the 10 traits used to compute the PCA, namely, antagonist activity against *Cmm*, *Pco*, *Pto*, *Xep*, and *Fol* (*Cmm*, *Pco*, *Pto*, *Xep*, and *Fol*), fluorescence on King’s medium B agar (Fluor), Gram reaction (Gram), phosphate solubilization (Phos_sol), salt tolerance (Salt_tol), and siderophore production (Sid_prod). **(C)** Heatmap of the pairwise correlations between the traits analyzed.

PCA (Figures 4A,B) was used to visualize the relationships between the 10 phenotypic traits analyzed (Gram reaction, fluorescence production, siderophore production, phosphate solubilization, salt tolerance, antagonist activity against *Cmm*, *Pco*, *Pto*, *Xep*, and *Fol*) of all bacterial isolates and the source of isolation (farm; root compartment). The first two principal components (PCs) explained 41% of the total phenotypic variability (PC1 = 25.3%, PC2 = 15.7%; Figures 4A,B). Results enabled the bacteria to be clearly separated according to the farm in which they were isolated (Figure 4A), but not to the root compartment (data not shown): bacteria collected on Farm 1 were mainly separated according to PC2, whereas bacteria from Farms 2 and 3 clustered mainly in the upper-right PCA quadrant (PC1 > 0, PC2 > 0); and bacteria collected from Farm 4 were mainly plotted in the lower-right (PC1 < 0, PC2 > 0) and lower-left (PC1 and PC2 < 0) PCA quadrants (Supplementary Table 2). The variables greatly influencing the bacteria disposition along the first two PCs were the antagonistic activity against *Pto*, *Pco*, and *Xep* and siderophore production. The antagonistic activity against *Pto* and the siderophore production highlighted opposite directions in the PCA biplot as they were oriented toward the upper-right quadrant and lower-left quadrants, respectively. On the other hand, the antagonist activity against *Xep* and *Pco* was oriented toward the lower-right PCA quadrant.

Overall, the 10 traits employed in the PCA showed a pairwise correlation ranging from -0.45 (*p*-value < 2.2–16) for siderophore production and antagonist activity against *Pto* to 0.34 (*p* < 1.1–12) for antagonist activity against *Xep* and *Pto* (Figure 4C). An ANOVA test using the collection farm and the 10 traits as categorical variables showed *p*-values that exceeded the significance threshold (*p* < 0.05) for all traits tested. The traits showing the highest significance (*p* < 0.0001) were the Gram reaction, siderophore production, and the antagonist activity against *Cmm*, *Pto*, *Pco*, and *Fol*.

**FIGURE 5:**
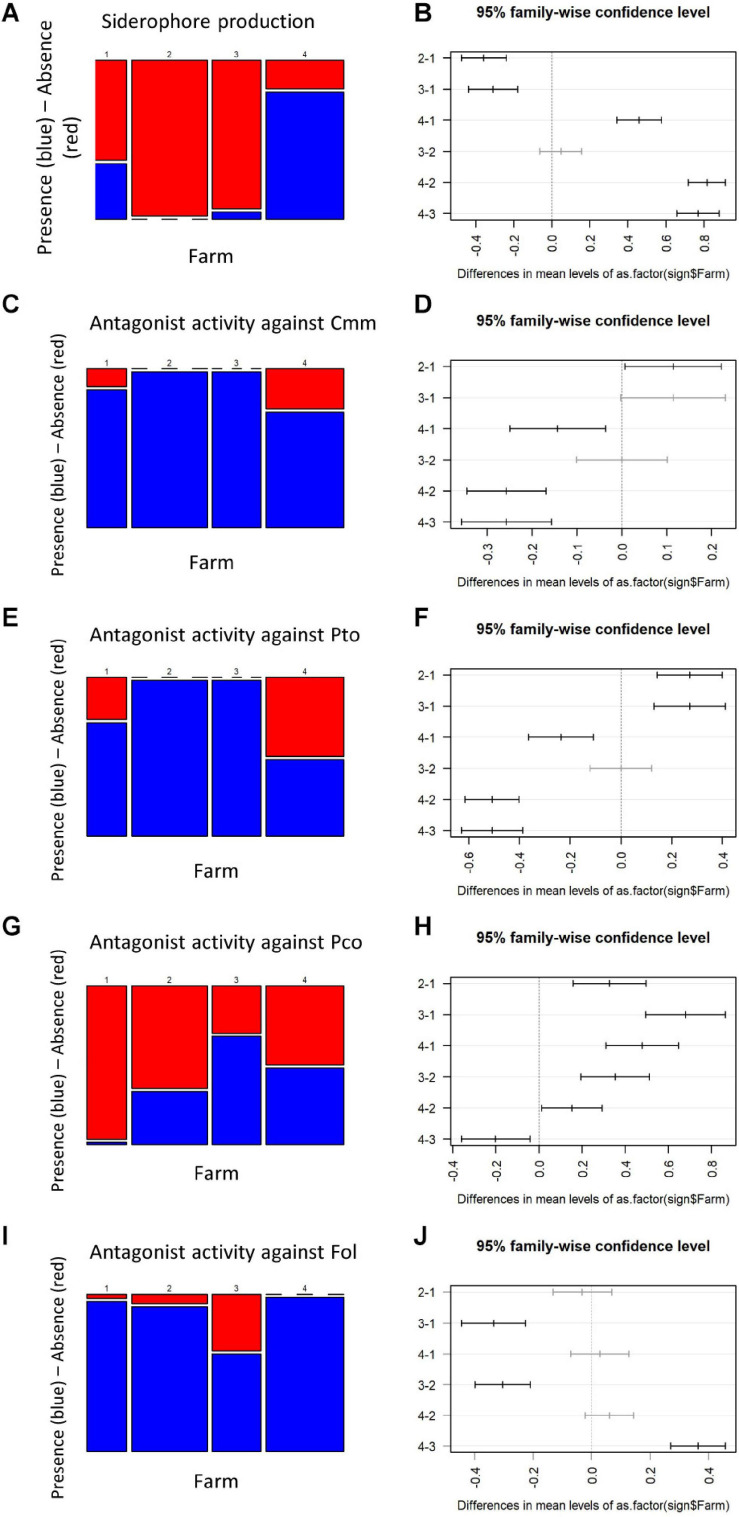
Mosaic plot and Tukey plot of the traits showing the highest differentiation among farms (ANOVA test, *p* < 0.0001), namely, siderophore production **(A)**, antagonistic activity against *Cmm* **(C)**, *Pto* **(E)**, *Pco* **(G)**, and *Fol* **(I)**. Mosaic plots [left of the panels **(B**,**D**,**F**,**H**,**J)**] show the relative frequency of the presence (blue) or absence (red) of a trait (*y*-axis) given the farm (*x*-axis); the width of the columns is proportional to the numerosity of the accessions isolated on each farm. On the right of the panels, the confidence intervals are shown of each pairwise comparison after the Tukey *post hoc* test. Pairwise comparisons that were not statistically different are shown in gray.

The bacteria distribution among the four farms was consistent for siderophore production and antagonist activity against *Cmm* and *Pto* (Figures 5A–J). Isolates collected from Farm 2 and Farm 3 were characterized by a substantial absence of siderophore production (Figures 5A,B) and positive antagonist activity against *Cmm* and *Pto* in all the samples (Figures 5C–F), whereas a more admixed configuration was registered for Farm 1 and Farm 4 (Figures 5C–F).

The *Pco* antagonistic activity showed statistical differences among all the four farms analyzed (Figures 5G,H), with bacteria collected on Farm 3 and Farm 1 showing the highest and lowest number of *Pco* antagonistic activity, respectively (Figure 5G). The antagonist activity against *Fol* was detected on all farms (Figures 5I,J).

### Bioprospecting of Tomato Endophytic Bacteria

Of the 100 total tomato root bacterial endophytes in the working collection, 77 were selected based on their phenotype and representativeness of the PGP and BCA traits, with at least two and/or three PGP traits and antagonistic activity to at least three microorganisms. Partial sequences of the 16S rRNA genes of the 77 isolates obtained from the E were analyzed. According to BLASTN similarity matches, isolates were identified by partial sequencing of their 16S rRNA gene, which enabled the isolates to be classified into three orders, namely, Bacillales, with all the bacterial isolates belonging to the genus *Bacillus*; Pseudomonadales, with bacterial isolates in the genera *Pseudomonas* and *Acinetobacter*; Enterobacteriales with isolates in the genera *Enterobacter*, *Ewingella*, *Pantoea*, *Providencia*, and *Lelliottia*. Putative single-isolate taxon is shown in Supplementary Table 3.

Four different *Bacillus* species were identified, three strains with 100% similarity to *Bacillus subtilis* (GenBank accession no CP051860.1, MT081484.1, KU729674.1); two strains with 100% similarity to *Bacillus amyloliquefaciens* (GenBank acc. no MK501609.1); 11 strains with 99–100% similarity to *B. velenzensis* (GenBank acc. no MN559711.1, CP051463.1, KY927398.1, MT365117.1, MN654121.1, and CP024922.1) (all species of the *B. subtilis* clade, Fan et al., 2017); one strain with 99% similarity to *B. megaterium* (GenBank accession no KT883839.1). Two strains were only identified at the genus level as *Bacillus* species (100% similarity to GenBank accession no. CP040881.1).

For isolates among the Enterobacteriales, the best hits were observed with the following species: 10 strains with 97% similarity to *Enterobacter cancerogenus* (GenBank accession no. FJ976582.1); one strain with 97% similarity to *Enterobacter tabaci* (GenBank accession no. MF682952.1); one strain with 97% similarity to *Enterobacter mori* (GenBank accession no. KJ589489.1); 10 strains were only identified at the genus level as *Lelliottia* (96–97% similarity to strain GenBank accession no. JN853247.1); three strains with 98–100% similarity to *Ewingella americana* (GenBank accession no. MT101745.1 and KY126991.1). Three strains with 99% similarity to *Providencia vermicola* (GenBank accession no. KX394623.1 and MK942706.1). Four strains were only identified at the genus level as *Pantoea* species (97% similarity to strains GenBank accession no. MK229045.1 and MH884045.1).

Different species were found in the genus *Pseudomonas* all within the *Pseudomonas putida* group within the *Pseudomonas fluorescens* lineage (Mulet et al., 2010): 14 strains with 100% similarity to *P. plecoglossicida* (GenBank accession no. MT367715.1); one strain with 100% similarity to *P. citronellolis* (GenkBank accession no. KM210226.1); one strain with 100% similarity to *P. monteilii* (GenkBank accession no. MH603875.1); four strains with 100% similarity to *P. putida* (GenkBank accession no. LN866622.1 and CP026115.2). All the isolates in the genus *Acinetobacter* showed the highest similarity to *Acinetobacter baumannii* (99–100% similarity to GenBank accession no. MT256198.1 and CP050388.1).

**FIGURE 6:**
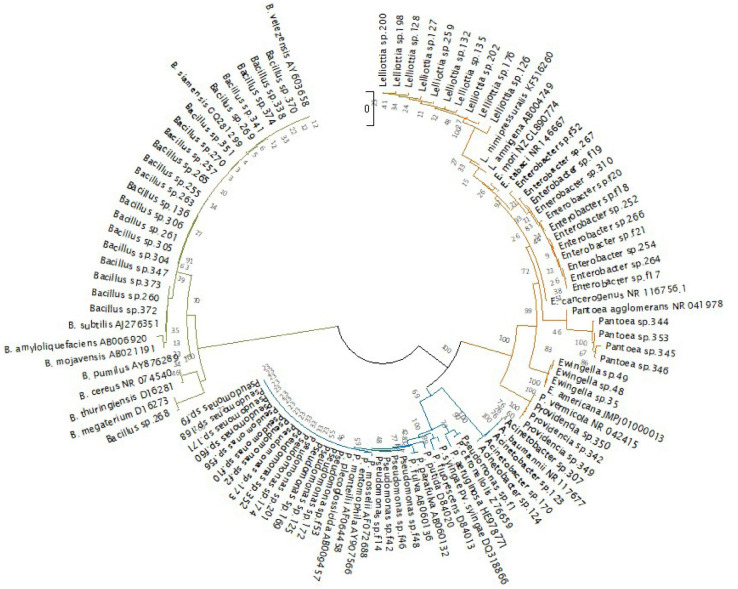
Phylogenetic tree of the 77 endophytic strains isolated in this study and 29 bacterial type strains. (*Bacillales* in green, *Pseudomonadales* in blue, and *Enterobacteriales* in orange). The evolutionary history was inferred using the neighbor-joining method (Saitou and Nei, 1987). The evolutionary distances were computed using the Kimura 2-parameter method (Kimura, 1980). There were a total of 824 positions in the final dataset. Evolutionary analyses were conducted in MEGA X (Kumar et al., 2018).

In the dendrogram showing the phylogenetic relationships of the endophytic strains in which type strains of the putative bacterial species and some reference species were included, the taxonomic position was confirmed, although some isolates clustered with appropriate the taxonomic clade (e.g., *B. subtilis* or *P. putida* clade) and not with the type strain of the bacterial species resulted from the BLAST similarity analysis. For this reason, sequences of the isolates were deposited at GenBank with the genus and strain name under accession numbers from MW130753 to MW130829 (Figure 6 and Supplementary Tables 3, 4).

**FIGURE 7:**
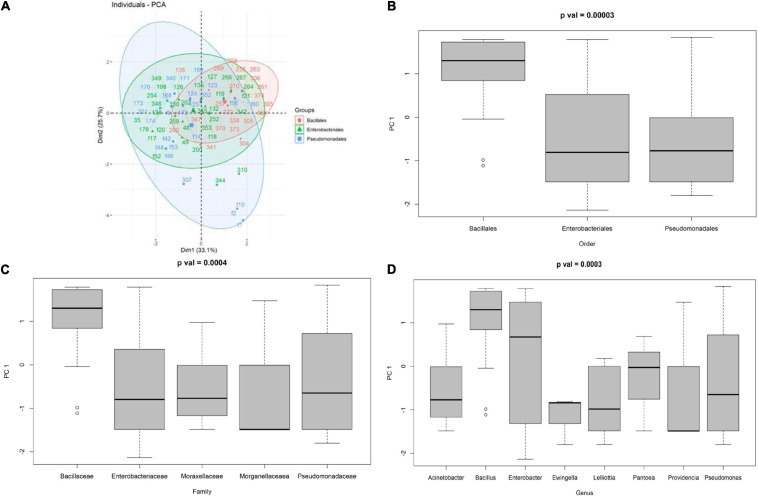
**(A)** PCA of the antagonist activity of the endophytic bacteria against *Cmm*, *Pco*, *Pto*, *Xep*, and *Fol*, boxplot of the distribution of the first principal component given the order **(B)**, family **(C)**, and genus **(D)**.

The PCA calculated on the 77 endophytic bacteria showing antagonist activity to at least one pathogen is shown in Figure 7A. The first two PCs accounted for 58.8% of the total phenotypic variability, with PC1 accounting for 33.1% and PC2 for 25.7%. Bacillales were mainly plotted in the upper-right quadrant of the PCA biplot (PC1 and PC2 > 0), whereas both Enterobacteriales and Pseudomonadales were mainly characterized by PC1 negative values (resulting in a high prevalence of bacteria plotted in the upper-left and lower-left PCA quadrants). The high effectiveness of PC1 in distinguishing between the Bacillales compared to the other two families was confirmed by the ANOVA test, which showed a *p* = 0.00003 (Figures 7B–D).

### In Planta Bioassays

Tomato seedlings treated by soil drenching with 10 bacterial strains belonging to the genus *Pseudomonas* and *Bacillus*, selected from the endorhizosphere isolated bacteria data set, showed an increase in plant height as compared to water treated control seedlings. Thirty days after the treatment with the bacterial strains, tomato seedlings were from 1.3 to 22% higher than the control plants. *Pseudomonas* strains f56 and f1 and *Bacillus* strain 306 and 261 significantly promoted plant height compared to untreated controls (*p* < 0.05) (Supplementary Table 5). Variable results were recorded for the other growth parameters that did not show a clear effect on plant weight (fresh and dry) and dry matter percentage (Supplementary Table 5).

Symptoms of bacterial canker, caused by *Cmm*, were first observed in the control plants 14 days postinoculation (dpi). They consisted in the unilateral wilting of one or more leaflets. Generalized wilting symptoms started at 21 dpi. Thirty days postinoculation of *Cmm*, the disease indexes of the plants treated with *P. citronellolis* strain f1 and *B. velenzensis* strain 265 were significantly lower (*p* < 0.05) than that of control plants treated with a water. Both antagonistic isolates also reduced the percentage of dead plants; these were for the *P. citronellolis* strain f1, *B. velenzensis* strain 265, and control plants, 0, 14.30, and 75%, respectively (Table 3). The values of the AUDPC, which records the progression of the disease, although were not significant statistically, were also lower (Table 3). The effect of the soil treatments with the tomato bacterial endophytes was also evaluated on the tomato leaf pathogen *Xep.* The occurrence of lesions on leaves was observed on positive control plants treated with water starting from 6 dpi. In fact, there were minute chlorotic spots that turned necrotic and expanded by 10 dpi when the data were recorded. Significant differences were observed between the endophyte-treated plants that showed fewer spots than control plants, although differences were observed between bacterial strains. A reduction of the diseases ranging from 30 to 80% was observed (Table 3).

**TABLE 3:** Results of the *in vivo* assays of the biocontrol activity of bacterial endophytes against the tomato bacterial pathogens *Clavibacter michiganensis* subsp. *michiganensis* (*Cmm*) and *Xanthomonas euvesicatoria* pv. *perforans* (*Xep*).

	**Cmm**			**Xep**	
**Bacterial strains**	**DI 30 dpi^a^**	**AUDPC**	**% of dead plants**	**n. spot/cm^2^ leaf area^b^**	**% reduction^c^**
*Pseudomonas plecoglossicida _*171	4.71*cd*	51.78d	84.70%	4.02*bc*	43.506.96
*P. plecoglossicida _*172	4.00*abc*	35.29*abc*	57.14%	1.46a	79.501.79
*Pseudomonas citronellolis_*f1	2.28a	15.86a	0.00%	4.88c	31.4014.76
*Pseudomonas monteilii_*f53	4.14*abc*	40.43*abc*	57.14%	2.99*abc*	57.911.14
*P. plecoglossicida_*f56	4.71*cd*	33.00*abc*	84.70%	4.88c	31.3112.39
*Bacillus velezensis _*261	3.28*abc*	24.86*abc*	14.30%	2.00*ab*	71.834.40
*B. velezensis_*263	5.00c	47.64*bc*	100%	2.16*ab*	69.616.53
*B. velezensis _*265	2.71*ab*	30.35*abc*	14.30%	1.30a	81.703.20
*Bacillus megaterium_*268	3.00*abc*	22.71*ab*	28.57%	2.70*ab*	61.965.67
*B. velezensis _*306	3.00*abc*	32.93*abc*	14.30%	2.06*ab*	70.976.82
Positive control	4.71*cd*	36.92*abc*	71.42%	7.11d	/

*^*a*^DI 30 dpi: disease index based on a 0–5-point disease scale 30 days post-inoculation. *^*b*^Disease severity recorded as number of spot/cm^2^ leaf area assessed 10 days post–Xep inoculation. ^*c*^Percentage reduction in lesion numbers per unit leaf area compared to the pathogen-only control ± standard error. Means in a column followed by the same letter are not significantly different according to Student–Newman–Keuls test at p ≤ 0.05. AUDPC, area under the disease progress curve. http://bioinformatics.psb.ugent.be/webtools/Venn/.**

## Discussion

The main aim of this study was to establish a collection of culturable tomato root–associated bacteria, as well as to bioprospect the natural diversity of root-associated bacterial communities under a real-world environment represented here by an intensive tomato cultivation area characterized by extraseasonal greenhouse production. Although in recent years the advances in next-generation omic technologies have led to the possibility of revealing plant-associated microbiomes, culture-dependent methods are still necessary to bioprospect natural diversity as a source of new tools for sustainable agriculture (Quiza et al., 2015; Lee et al., 2016; Müller et al., 2016).

In this study, bacterial population sizes of the total numbers of fluorescent and spore-forming bacteria associated with the root environment of greenhouse tomato plants grown in agricultural soils from four different farms varied according to the compartment of isolation (i.e., rhizosphere, rhizoplane, endosphere) and, in some cases, the farms. The characterization of a collection of 424 bacterial isolates targeted at phenotypic traits (plant growth promotion and/or biocontrol of detrimental plant pathogens) did not show any clear relationships between the compartments of root isolation. In contrast, the isolates clustered according to the four isolation farms.

The four farms from where the samples were selected shared some common features of the cultivation area. Sicily is the principal tomato greenhouse production area in Italy, and more than half of the tomato production comes from the Province of Ragusa, where the four farms were located. This area is characterized by sandy soil and climatic conditions that facilitate out-of-season production. Tomatoes are grown for one or two cycles within the year in greenhouses covered with a plastic film. In the four greenhouses, four different genotypes of tomato were cultivated, which differed according to the farm management (irrigation, fertilization, and pesticide use and agronomic operations).

Overall, the phenotyping of 424 bacterial isolates from the tomato root environment revealed that this community was more represented by Gram-negative than Gram-positive bacteria and that they possessed interesting PGP bacterial traits. In fact, 139 of the 424 root-associated bacteria isolates were able to produce siderophores, solubilize phosphates, and grow on a saline medium.

These characteristics could be of great interest in developing bioinoculant with also biofertilizer abilities that could also promote plant growth and yield. Phosphate-solubilizing microorganisms play an important role in supplementing phosphorus to the plants, allowing a sustainable use of phosphate fertilizers. P-solubilizing activity is related to the microbial production of organic acids, which chelate the cation bound to phosphate, thereby converting it to a soluble form (Sagoe et al., 1998; Rashid et al., 2004; Lugtenberg and Kamilova, 2009). The ability to produce secondary metabolites such as siderophore and antimicrobial peptides has been evaluated in many studies on PGPRs. The ability to produce siderophore and metabolites contributing to antibiosis has been the focus of many studies on PGPR (Sayyed et al., 2004; Maksimov et al., 2011).

The high number of salinity-tolerant bacterial isolates suggests that a selection may have occurred as salinity is one of the typical characteristics of the area (i.e., soils of all the farms showed an EC > 2.0 mmhos cm^–1^ and high Na content). Tomato is moderately sensitive to salinity saline water that is used in greenhouse cultivations; however, high salinity may affect plant physiology (Leonardi and Martorana, 2005). Several reports have shown that halotolerant PGPRs improve the growth of various agricultural crops under salinity stress. Inoculating crops with halotolerant PGPRs isolated from halophytes has been successful in improving crop growth and tolerance under salt stress conditions (Shukla et al., 2012; Khan et al., 2016).

More importantly, approximately 30% (129 strains) of the root-associated bacterial isolates showed antagonistic activity against all the five tested phytopathogens, although to different extents. Their antagonistic activity as assessed *in vitro* suggests the production of secondary metabolites with inhibitory activity against fungi and Gram-positive and Gram-negative plant pathogenic bacteria. Some of these harmful pathogen are seed and/or soil transmitted (Bardin and Gullino, 2020; Catara and Bella, 2020).

A large number of studies have shown that tomato bacterial communities, resolved by metagenomics based on amplicon sequencing, are influenced by different factors. Data, however, refer to the taxonomic operational units, and the PGP and BC activities can only be inferred. Among the biotic and abiotic factors, soil is considered the primary force driving plant–microbiota diversity (Jeanbille et al., 2016). Different studies have demonstrated that the influence of the soil plays a stronger role on plant–microbiota diversity than the plant genotype (Poli et al., 2016; Dong et al., 2019). In addition, transcriptomics and proteomics have demonstrated that the overall characteristics of the substrate contribute more than plant genotype to shaping the molecular responses in tomato roots (Chialva et al., 2018).

Our research also focused on bacterial endophytes, which are good candidates for beneficial inoculants aimed at reducing the chemical inputs in conventional agricultural practices and increasing nutrient uptake and stress resilience in plant species (Ryan et al., 2008; Gupta et al., 2014). In fact, the endophytes interact more closely with their host than rhizospheric bacteria because they are located within the plant tissues (Hallmann et al., 1997; Weyens et al., 2013). In addition, as they live in the apoplast (or in the xylem), endophytes do not need to compete for nutrition and/or niche in the soil as bacteria do in the rhizosphere (Reiter et al., 2002), and they or their metabolites can easily reach the pathogens within the plants (Gupta et al., 2014).

A subset of bacterial endophytes isolated from tomato endorhizosphere (77 isolates), identified by partial sequencing of their 16S rRNA gene, belonged to two phyla (Firmicutes and Proteobacteria) and to three orders, namely, Bacillales (27.3%), with all the isolates in the genus *Bacillus*; Pseudomonadales (31.2%), with isolates in the genera *Pseudomonas* and *Acinetobacter*; and Enterobacteriales (41.6%), with isolates in the genera *Enterobacter*, *Ewingella*, *Pantoea*, *Providencia*, and *Lelliottia*. Similarly, some of these genera (*Bacillus*, *Pseudomonas*, *Acinetobacter*, *Enterobacter*) have been isolated from tomato endorhizosphere in studies on beneficial bacteria (Tian et al., 2017; Singh et al., 2019). Bacterial strains in the genera, *Rhizobium* and *Ralstonia*, have also been isolated from the endorhizophere of tomato plants (Abbamondi et al., 2016). Bergna et al. (2018) isolated *Ralstonia*, *Stenotrophomonas*, and *Bacillus* strains both from tomato root and seed endosphere. The high-throughput screening and cultivable approach suggested that beneficial bacteria are seed transmitted (Bergna et al., 2018).

Recent studies demonstrated that tomato bacterial communities of the root zone and of the rhizosphere exhibited the highest richness and diversity in comparison to those of the endorhizosphere (Dong et al., 2019; Lee et al., 2019). In general, the richness decreased from the root zone soil to rhizosphere to phyllosphere to endosphere, whereas the diversity decreased in a different order: root zone soil > rhizosphere > endosphere > phyllophere (Ottesen et al., 2013; Dong et al., 2019). Our results, however, suggest that beneficial activities are commonly spread in each root compartment. However, the richness of the bacterial community is the lowest in the endorhizosphere. In this study, when the relationship between the bacterial families and the antagonistic activity of the tomato endophytes was investigated, *Bacillus* isolates were significantly more antagonistic *in vitro* against tomato plant pathogens than bacterial isolates belonging to Pseudomonadales and Enterobacteriales.

Almost 40% of the endophytic bacteria characterized here belong to Enterobacteriales, more specifically to the Enterobacteriaceae family, and as many as five different genera were recorded. Many studies have confirmed that Enterobacteriaceae are indigenous components of the plant microbiome in different species (Brandl, 2006; Teplitski et al., 2011; Erlacher et al., 2014, 2015; Tian et al., 2017). Data on rocket salad suggested that the soil probably provides the largest reservoir from which enterics become established and spread within the whole plant (Cernava et al., 2019). Enterobacteriaceae have been successfully evaluated as biocontrol agents in tomato (Xue et al., 2009); however, there are still some concerns regarding the use of these antagonistic bacteria as some species are human pathogens (Erlacher et al., 2015; Cernava et al., 2019).

Some *Pseudomonas* species show considerable potential for the suppression of plant pathogens, in promoting plant growth, inducing systemic resistance in plants, and are widely used as biocontrol agents (Mercado-Blanco and Bakker, 2007). These bacteria produce several diffusible and/or volatile secondary metabolites with antibiotic properties such as diacetylphloroglucinol, pyrrolnitrin, and cyclic lipopeptides phenazine (Haas and Keel, 2003; Raaijmakers and Mazzola, 2012).

Most endophytic *Bacillus* isolates identified here with the 16S rRNA gene sequence belong to the *B. amyloliquefaciens* and *B. subtilis* group. Members of the *B. subtilis* species complex, which includes at present more than 20 closely related species such as *B. subtilis*, *B. amyloliquefaciens*, and *Bacillus pumilus* and, to a lesser extent, the genus *Paenibacillu*s species, have been proven to be efficient at plant growth promotion and biocontrol against plant pathogens, such as viruses, bacteria, fungi, and nematodes, in the vicinity of plant roots (Vacheron et al., 2013; Fan et al., 2017). To date, bacilli are the most widely used bacteria on the biopesticide market (Borriss, 2011, 2015). This is mainly due to their ability to produce durable endospores, which enable stable bioformulations to be prepared with a long shelf life. These antagonistic strains produce numerous antibiotics including polymyxin, difficidin, subtilin, mycobacillin, and zwittermicin A, which are active against plant pathogenic bacteria and fungi (Borriss, 2015; Caulier et al., 2019).

Biological control of a set of *Bacillus* and *Pseudomonas* isolates from tomato endorhizosphere was tested in a growth chamber in two separate experiments. Two important bacterial pathogens that are common in the area of sampling and that represent important seed-transmitted pathogens were chosen: (i) the vascular pathogenic bacterium *C. michiganensis* pv. *michiganensis*, which causes tomato bacterial canker; and (ii) one of the *Xanthomonas* species that causes bacterial spot of tomato, *X. euvesicatoria* pv. *perforans* (Bella et al., 2012; Aiello et al., 2013; Catara and Bella, 2020).

Biocontrol of bacterial pathogens reduces the impact of copper compounds. Our results were encouraging as in the growth chamber *Cmm* spread very quickly inside the plantlets. In fact, 1 month after inoculation, *Cmm* led to the death of 100% of the plants in the control. Two isolates, *Pseudomonas* species f1 and *Bacillus* species, 265 out of the 10 bacterial isolates tested *in vivo*, significantly reduced bacterial canker by delaying disease progress and reducing the number of dead plants at the end of the trial compared to the control. Several studies have shown that *Pseudomonas* or *Bacillus* strains inoculated in the soil or in the seeds can reduce the incidence and severity of bacterial canker, and in some cases, an induction of systemic resistance has been suggested (Boudyach et al., 2004; Nandi et al., 2018; Abo-Elyousr et al., 2019). In our pathogenicity tests, we used two different pathosystems. In the biocontrol trial on bacterial canker, both the pathogenic bacterium and the biocontrol agent have been inoculated in the soil where the two microorganisms may have interacted by competition and/or antibiotic phenomena. All biocontrol bacteria tested were able to reduce the symptoms of bacterial spot. As the phytopathogenic bacterium in this case was inoculated on the leaves, the spatial distance between the two suggests that the mechanism of action also involves induction of systemic resistance. Phage therapy is currently considered the most effective *Xep* biological control method (Obradovic et al., 2005). However, the effect of foliar biocontrol bacteria and PGPRs and *B. pumilus* in reducing bacterial spot in greenhouse and some field trials has been already demonstrated (Byrne et al., 2005; Ji et al., 2006).

The microbial collection generated in this study could provide the basis for the future development of bioinoculants using single strains or synthetic microbial communities. The bacterial isolates were obtained from the same niche of pathogens; thus, it is conceivable that they could colonize tomato roots, although endophytic colonization is still to be demonstrated. The use of microbial consortia has recently emerged as an approach to combine microorganisms with different traits, effects, or mechanisms of action (Compant et al., 2019). Future *in vivo* studies will demonstrate how successful this bottom-up approach is and whether the isolates could be used to inoculate plantlets in the nursery, thus providing intensive tomato cultivation areas with protected plants.

## Data Availability Statement

Sequences of the isolates used in the present study were submitted to the GenBank database. GenBank accession numbers (MW130753–MW130829) are shown in Supplementary Table 3.

## Author Contributions

AA designed and performed all the experiments and wrote the manuscript together with VC. GD and PB contributed to perform 16S rRNA gene amplification, sequencing, and analysis. MD critically contributed to data organization and performed data analysis. RZ planned farm selection and soil analysis and provided sampling. FG, GD, and AA performed the *in vivo* experiments. GC contributed to the critical analysis of the results and together with MD, VC, and PB contributed to the writing and revision of the manuscript. VC conceived, organized, and supervised the project. All authors contributed to the article and approved the submitted version.

## Conflict of Interest

The authors declare that the research was conducted in the absence of any commercial or financial relationships that could be construed as a potential conflict of interest.

## Footnotes

1 http://faostat.fao.org, accessed September 20, 2020.

2 http://www.ncbi.nlm.nih.gov
