# Supplementary material for: Bioprospecting of Beneficial Bacteria Traits Associated With Tomato Root in Greenhouse Environment Reveals That Sampling Sites Impact More Than the Root Compartment
Source: Front Plant Sci. 2021 Apr 13;12:637582. doi: 10.3389/fpls.2021.637582 (PMC8078776; doi:10.3389/fpls.2021.637582)
Supplement: Supplementary file 1 [file Data_Sheet_1.PDF]

Supplementary Material

Bioprospecting of beneficial bacteria traits associated with tomato root in greenhouse environment reveals that sampling sites impact more than the root compartment.

Alice Anzalone[1], Mario Di Guardo[1], Patrizia Bella[2], Farideh Ghadamgahi[3], Giulio Dimaria[1], Rosario Zago[3], Gabriella Cirvilleri[1], Vittoria Catara[1*]

*** Correspondence:**
vcatara@unict.it

1 Supplementary Figures and Tables

1.1 Supplementary Figures

1.1.1 Figure S1. Heat map obtained using two arbitrary scales to quantitative evaluate the antagonistic potential bacterial collected according to the farms and root compartment of isolation (E, endorhizosphere, RP, rhizoplane and, R, rhizosphere). For a quantitative evaluation of the bacterial strains antagonistic activity two arbitrary 0-3 scales were used. The antibacterial activity was scored based on the growth inhibition area size as: 0, no antagonism; 1, < 3 mm; 2, ≥ 3, <10 mm; 3, >10 mm. Antifungal activity was scored based on the percentage of growth inhibition against *Fol* (PGI) as follow: 0, no inhibition, 1, PGI <30%; 2, PGI 30- 60%; 3, PGI >60%.

1.2 Supplementary Table

1.2.1 Table S1: Phenotypic qualitative evaluation of the presence (1) or absence (0) of beneficial traits of bacteria isolated from rhizosphere (R), rhizoplane (RP), and endorhizosphere (E) .

1.2.2 Table S2: Principal Component Analysis (PCA) table of the full dataset on 424 bacteria, showing relationships between phenotypic traits (Gram reaction, fluorescence production, siderophore production, phosphate solubilisation, salt tolerance, antagonist activity against *Cmm*, *Pco*, *Pto*, *Xep*, and *Fol*) and bacterial isolates.

1.2.3 Table S3: Molecular identification of the endophytic bacteria based on the 16S rRNA gene sequence.

1.2.4 Table S4: Accession numbers of the sequences of the type strains used as references in the dendrogram in figure 6 in this study.

1.2.5 Table S5: Effect of the treatments by soil drenching of tomato plantlets grown in pots using bacterial endophytes belonging to the genus *Pseudomonas* and *Bacillus*. Plant height, fresh and dry weight of roots and shoots, dry matter and root/shoot ratio was measured 30 days after the treatment.

Figure S1: Heat map obtained using two arbitrary scales to quantitative evaluate the antagonistic potential bacterial collected according to the farms and root compartment of isolation (E, endorhizosphere, RP, rhizoplane and, R, rhizosphere). For a quantitative evaluation of the bacterial strains antagonistic activity two arbitrary 0-3 scales were used. The antibacterial activity was scored based on the growth inhibition area size as: 0, no antagonism; 1, < 3 mm; 2, ≥ 3, <10 mm; 3, >10 mm. Antifungal activity was scored based on the percentage of growth inhibition against *Fol* (PGI) as follow: 0, no inhibition, 1, PGI <30%; 2, PGI 30- 60%; 3, PGI >60%.

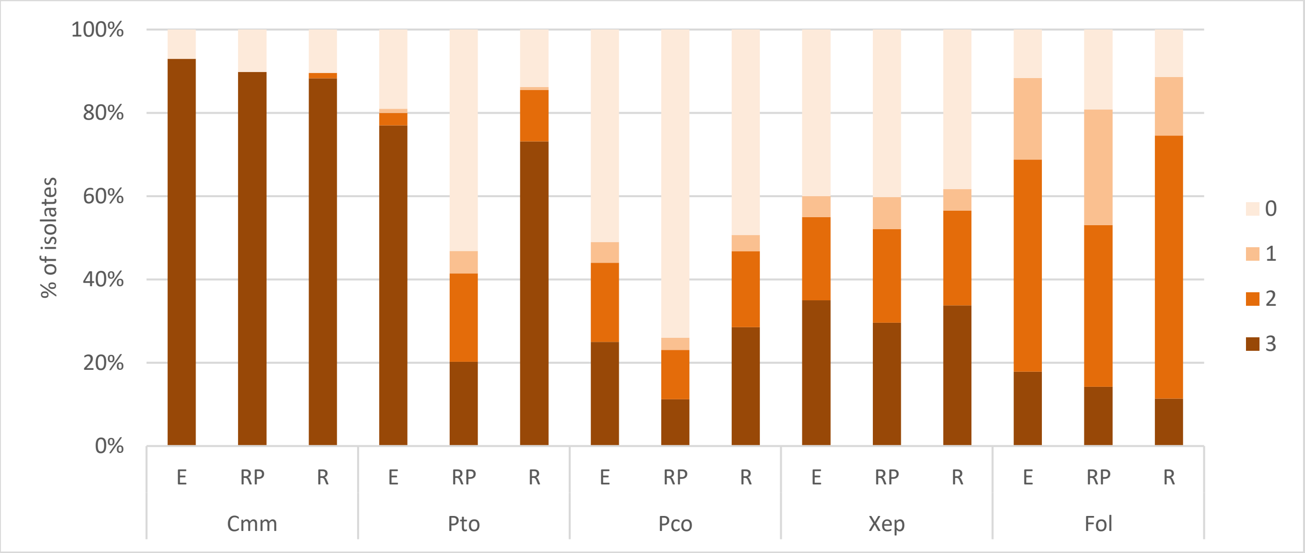

Table S1: Phenotypic qualitative evaluation of the presence (1) or absence (0) of beneficial traits of bacteria isolated from rhizosphere (R), rhizoplane (RP), and endorhizosphere (E).

Farm	Root compartment	Name ID	Gram	Fluorescence	PGP activity			Antagonistic activity				
					Siderophore production	Phosphate solubilization	Salt tolerance (NaCl 8%)	*Cmm*	*Pto*	*Pco*	*Xep*	*Fol*
**1**	RP	**1**	0	0	1	0	0	1	1	0	1	1
**1**	RP	**2**	0	0	1	0	1	1	1	0	1	1
**1**	RP	**3**	0	1	1	0	1	1	0	0	1	1
**1**	RP	**4**	0	0	1	0	1	1	0	0	1	1
**1**	RP	**5**	0	0	1	0	1	1	0	0	1	1
**1**	RP	**6**	0	0	1	0	1	1	0	0	1	1
**1**	RP	**7**	0	0	1	0	1	1	1	0	1	1
**1**	RP	**8**	0	0	1	0	0	1	1	0	1	1
**1**	RP	**9**	0	0	1	0	1	1	0	0	1	1
**1**	RP	**10**	0	0	0	0	1	1	0	0	1	1
**1**	RP	**11**	0	0	0	0	1	1	1	0	1	1
**1**	RP	**12**	0	0	0	0	1	1	0	0	1	1
**1**	RP	**13**	0	0	0	0	1	1	1	0	1	1
**1**	RP	**14**	0	0	0	0	1	1	0	0	0	1
**1**	RP	**15**	0	0	0	0	1	1	0	0	0	1
**1**	RP	**16**	0	0	1	0	1	1	1	0	0	1
**1**	RP	**17**	0	0	0	0	1	1	0	0	0	1
**1**	RP	**18**	0	0	0	0	1	1	1	0	0	1
**1**	R	**19**	0	0	0	0	1	1	1	0	0	1
**1**	RP	**20**	0	0	0	0	1	1	1	0	1	1
**1**	RP	**21**	0	0	0	0	1	1	1	0	1	1
**1**	RP	**22**	0	0	0	0	1	1	1	0	1	1
**1**	RP	**23**	0	0	0	0	1	1	1	0	1	1
**1**	RP	**24**	0	0	0	0	1	1	1	0	1	1
**1**	RP	**25**	0	0	1	0	1	0	1	0	1	0
**1**	RP	**26**	0	0	1	0	1	0	0	0	0	0
**1**	RP	**27**	0	0	0	0	1	1	1	0	1	1
**1**	RP	**28**	0	1	0	0	1	1	1	0	1	1
**1**	RP	**29**	0	0	0	0	1	1	1	0	1	1
**1**	RP	**30**	0	0	0	0	1	1	1	0	1	1
**1**	RP	**31**	0	0	0	0	1	0	0	0	0	1
**1**	RP	**32**	0	0	0	0	1	0	0	0	0	1
**1**	RP	**33**	0	0	0	0	1	1	0	0	0	1
**1**	RP	**34**	0	0	0	0	1	1	0	0	0	1
**1**	RP	**35**	0	1	0	0	1	1	0	0	0	1
**1**	RP	**36**	0	0	0	0	1	1	1	0	1	1
**1**	RP	**37**	0	1	0	0	1	1	1	0	1	1
**1**	RP	**38**	0	0	0	0	1	1	0	0	0	1
**1**	RP	**39**	0	0	0	0	1	1	1	0	1	1
**1**	RP	**40**	0	0	0	0	1	0	1	0	1	1
**1**	RP	**41**	0	0	0	0	1	1	1	0	1	1
**1**	RP	**42**	0	0	1	0	1	1	1	0	1	1
**1**	RP	**43**	0	0	1	0	1	1	1	0	1	1
**1**	RP	**44**	0	0	1	0	1	1	1	0	1	1
**1**	RP	**45**	0	0	0	0	1	1	1	0	1	1
**1**	RP	**46**	0	0	0	0	1	1	1	0	1	1

Farm	Root compartment	Name ID	Gram	Fluorescence	PGP activity			Antagonistic activity				
					Siderophore production	Phosphate solubilization	Salt tolerance (NaCl 8%)	*Cmm*	*Pto*	*Pco*	*Xep*	*Fol*
**1**	RP	**47**	0	0	1	0	1	1	1	0	1	1
**1**	E	**48**	0	0	1	0	1	1	1	0	1	1
**1**	E	**49**	0	0	0	1	1	1	0	0	1	1
**1**	R	**50**	0	0	0	1	1	1	1	0	1	1
**1**	R	**51**	0	0	0	1	1	1	0	0	1	1
**1**	R	**52**	0	0	1	1	1	1	1	0	1	1
**1**	R	**53**	0	0	1	1	1	1	1	0	1	1
**1**	R	**54**	0	0	1	1	1	1	1	0	1	1
**1**	R	**55**	0	0	1	1	1	1	1	0	1	1
**1**	R	**56**	0	0	1	1	1	1	1	1	1	1
**1**	R	**57**	0	0	0	1	1	1	1	0	1	1
**1**	R	**58**	0	0	0	1	1	1	1	0	1	1
**1**	R	**59**	0	0	0	1	0	1	1	0	1	1
**1**	R	**60**	0	0	0	1	0	1	1	0	1	1
**1**	R	**61**	0	0	0	1	1	1	1	0	1	1
**1**	R	**62**	0	0	0	1	0	1	1	0	1	1
**1**	RP	**63**	0	0	0	1	0	1	1	0	1	1
**1**	RP	**64**	0	0	1	1	0	1	1	0	1	1
**1**	RP	**65**	0	0	1	0	0	1	1	0	0	1
**1**	RP	**66**	0	0	0	0	0	1	1	0	0	1
**1**	R	**67**	0	0	1	1	0	0	1	0	0	1
**1**	R	**68**	0	0	0	1	0	0	1	0	0	1
**1**	R	**69**	0	0	0	1	0	1	1	0	0	1
**1**	R	**70**	0	0	0	1	0	0	1	0	0	1
**2**	R	**91**	0	0	0	1	1	1	1	0	0	1
**2**	R	**92**	0	0	0	1	1	1	1	1	1	1
**2**	R	**93**	0	0	0	0	1	1	1	1	1	1
**2**	R	**94**	1	0	0	0	1	1	1	1	0	1
**2**	R	**95**	0	0	0	0	1	1	1	1	1	1
**2**	R	**96**	0	0	0	1	1	1	1	1	0	1
**2**	R	**97**	1	0	0	1	0	1	1	1	1	1
**2**	R	**98**	0	0	0	1	0	1	1	1	1	1
**2**	R	**99**	1	0	0	1	1	1	1	1	1	1
**2**	R	**100**	0	0	0	0	1	1	1	0	1	1
**2**	R	**101**	0	0	0	0	0	1	1	0	1	1
**2**	R	**102**	0	0	0	0	0	1	1	1	0	1
**2**	R	**103**	0	0	0	0	1	1	1	0	1	1
**2**	R	**104**	0	0	0	0	1	1	1	1	1	1
**2**	R	**105**	0	0	0	1	1	1	1	1	1	1
**2**	R	**106**	0	0	0	0	0	1	1	0	0	1
**2**	RP	**107**	0	0	0	1	1	1	1	0	1	1
**2**	RP	**108**	0	0	0	0	0	1	1	0	1	1
**2**	RP	**109**	0	0	0	0	0	1	1	0	0	1
**2**	RP	**110**	0	0	0	0	1	1	1	0	0	1
**2**	RP	**111**	0	0	0	0	1	1	1	0	1	1
**2**	RP	**112**	1	0	0	0	1	1	1	0	0	1
**2**	RP	**113**	0	0	0	0	1	1	1	0	1	1
**2**	R	**114**	0	0	0	0	1	1	1	0	1	1

Farm	Root compartment	Name ID	Gram	Fluorescence	PGP activity			Antagonistic activity				
					Siderophore production	Phosphate solubilization	Salt tolerance (NaCl 8%)	*Cmm*	*Pto*	*Pco*	*Xep*	*Fol*
**2**	RP	**115**	0	0	0	1	1	1	1	1	0	1
**2**	RP	**116**	0	0	0	0	1	1	1	1	1	1
**2**	RP	**117**	0	0	0	0	1	1	1	1	1	1
**2**	RP	**118**	0	0	0	0	0	1	1	0	1	1
**2**	RP	**119**	0	0	0	0	1	1	1	0	1	1
**2**	RP	**120**	0	0	0	0	1	1	1	0	1	1
**2**	RP	**121**	1	0	0	0	1	1	1	0	0	0
**2**	RP	**122**	0	0	0	1	1	1	1	0	0	0
**2**	E	**123**	0	0	0	1	0	1	1	1	1	1
**2**	E	**124**	0	0	0	0	1	1	1	1	1	1
**2**	E	**125**	0	0	0	0	1	1	1	1	1	1
**2**	E	**126**	0	0	0	0	1	1	1	0	1	1
**2**	E	**127**	0	0	0	0	0	1	1	1	1	1
**2**	E	**128**	0	0	0	0	0	1	1	0	0	1
**2**	E	**129**	0	0	0	0	1	1	1	0	0	0
**2**	E	**130**	1	0	0	0	1	1	1	0	0	0
**2**	E	**131**	0	0	0	1	0	1	1	1	1	1
**2**	E	**132**	0	0	0	0	1	1	1	1	1	1
**2**	E	**133**	0	0	0	0	1	1	1	0	0	1
**2**	E	**134**	0	0	0	0	1	1	1	1	1	1
**2**	E	**135**	1	0	0	0	0	1	1	1	1	1
**2**	E	**136**	1	0	0	0	1	1	1	0	1	1
**2**	R	**137**	1	0	0	0	1	1	1	0	1	1
**2**	R	**138**	0	0	0	1	1	1	1	1	1	1
**2**	R	**139**	1	0	0	0	1	1	1	1	1	1
**2**	R	**140**	0	0	0	0	1	1	1	1	1	1
**2**	R	**141**	1	0	0	1	1	1	1	0	1	1
**2**	R	**142**	0	0	0	1	0	1	1	0	0	1
**2**	R	**143**	1	0	0	1	1	1	1	0	1	1
**2**	R	**144**	0	0	0	0	0	1	1	1	1	0
**2**	R	**145**	1	0	0	0	0	1	1	0	0	1
**2**	R	**146**	0	0	0	0	0	1	1	0	1	1
**2**	R	**147**	0	0	0	1	1	1	1	0	1	1
**2**	R	**148**	1	0	0	1	1	1	1	0	1	1
**2**	R	**149**	0	0	0	1	1	1	1	0	1	1
**2**	R	**150**	1	0	0	1	1	1	1	0	1	1
**2**	R	**151**	1	0	0	1	0	1	1	1	1	1
**2**	R	**152**	1	0	0	0	1	1	1	0	1	1
**2**	R	**153**	1	0	0	0	1	1	1	0	0	1
**2**	R	**154**	1	0	0	0	1	1	1	1	1	1
**2**	R	**155**	0	0	0	1	1	1	1	1	1	1
**2**	R	**156**	1	0	0	0	1	1	1	0	0	1
**2**	R	**157**	1	0	0	0	1	1	1	1	1	1
**2**	R	**158**	1	0	0	0	1	1	1	1	0	1
**2**	R	**159**	1	0	0	0	1	1	1	0	0	1
**2**	RP	**160**	1	0	0	0	1	1	1	0	1	1
**2**	RP	**161**	1	0	0	0	0	1	1	0	1	1
**2**	RP	**162**	1	0	0	0	1	1	1	1	1	1
**2**	RP	**163**	1	0	0	1	1	1	1	0	1	1

Farm	Root compartment	Name ID	Gram	Fluorescence	PGP activity			Antagonistic activity				
					Siderophore production	Phosphate solubilization	Salt tolerance (NaCl 8%)	*Cmm*	*Pto*	*Pco*	*Xep*	*Fol*
**2**	RP	**164**	1	0	0	1	1	1	1	0	0	1
**2**	RP	**165**	0	0	0	0	1	1	1	1	1	1
**2**	RP	**166**	1	0	0	0	1	1	1	1	1	1
**2**	RP	**167**	1	0	0	0	1	1	1	0	1	1
**2**	E	**168**	0	0	0	0	1	1	1	0	0	1
**2**	E	**169**	0	0	0	0	1	1	1	0	1	1
**2**	E	**170**	1	0	0	0	1	1	1	0	0	1
**2**	E	**171**	1	0	0	1	1	1	1	0	1	1
**2**	E	**172**	1	0	0	1	1	1	1	0	1	1
**2**	E	**173**	1	0	0	0	1	1	1	0	0	1
**2**	E	**174**	1	0	0	1	1	1	1	0	0	1
**2**	E	**175**	1	0	0	1	1	1	1	0	1	1
**2**	E	**176**	0	0	0	1	1	1	1	0	0	1
**2**	R	**177**	1	0	0	1	1	1	1	0	0	1
**2**	R	**178**	1	0	0	1	1	1	1	0	0	1
**2**	R	**179**	1	0	0	1	1	1	1	1	1	1
**2**	R	**180**	0	0	0	1	1	1	1	0	1	1
**2**	R	**181**	0	0	0	0	1	1	1	0	0	1
**2**	R	**182**	0	0	0	1	1	1	1	1	0	1
**2**	RP	**183**	0	0	0	1	1	1	1	0	1	1
**2**	RP	**184**	0	0	0	1	1	1	1	0	1	1
**2**	RP	**185**	0	0	0	1	1	1	1	1	1	1
**2**	RP	**186**	0	0	0	1	1	1	1	1	1	1
**2**	RP	**187**	1	0	0	1	1	1	1	0	0	0
**2**	RP	**188**	0	0	0	1	1	1	1	0	1	1
**2**	RP	**189**	0	0	0	1	1	1	1	0	1	1
**2**	RP	**190**	0	0	0	1	1	1	1	0	1	1
**2**	RP	**191**	0	0	0	1	1	1	1	1	1	1
**2**	RP	**192**	1	0	0	1	1	1	1	0	1	1
**2**	RP	**193**	0	0	0	1	1	1	1	0	1	1
**2**	RP	**194**	1	0	0	1	1	1	1	0	0	1
**2**	RP	**195**	0	0	0	1	1	1	1	0	0	1
**2**	E	**196**	0	0	0	1	1	1	1	0	1	1
**2**	E	**197**	0	0	0	1	1	1	1	0	0	1
**2**	E	**198**	0	0	0	1	1	1	1	0	0	1
**2**	E	**199**	0	0	0	1	1	1	1	0	0	1
**2**	E	**200**	0	0	0	1	1	1	1	0	1	1
**2**	E	**201**	1	0	0	1	1	1	1	0	0	1
**2**	E	**202**	0	0	0	1	1	1	1	0	1	1
**2**	E	**203**	1	0	0	1	1	1	1	1	1	0
**2**	E	**204**	0	0	0	1	1	1	1	1	1	0
**2**	E	**205**	0	0	0	1	1	1	1	1	1	1
**2**	E	**206**	0	0	0	1	1	1	1	0	0	1
**2**	E	**207**	1	0	0	1	0	1	1	0	0	1
**2**	E	**208**	0	0	0	1	0	1	1	1	1	1
**2**	RP	**F11**	0	0	0	1	1	1	1	0	0	1
**2**	R	**F13**	0	0	0	1	1	1	1	0	1	1
**2**	E	**F1**	0	1	0	1	1	1	1	1	0	1

Farm	Root compartment	Name ID	Gram	Fluorescence	PGP activity			Antagonistic activity				
					Siderophore production	Phosphate solubilization	Salt tolerance (NaCl 8%)	*Cmm*	*Pto*	*Pco*	*Xep*	*Fol*
**2**	E	**F2**	0	1	0	1	1	1	1	0	1	1
**2**	RP	**F3**	0	1	0	1	0	1	1	0	0	1
**2**	RP	**F4**	0	1	0	1	1	1	1	0	0	1
**2**	RP	**F5**	0	1	0	1	1	1	1	0	0	1
**2**	RP	**F6**	0	1	0	1	1	1	1	0	0	1
**2**	R	**F7**	0	0	0	1	1	1	1	0	0	1
**2**	R	**F8**	0	0	0	1	1	1	1	0	0	1
**2**	E	**F9**	0	1	0	1	0	1	1	0	1	1
**2**	E	**F10**	0	1	0	1	0	1	1	0	1	1
**2**	RP	**F12**	0	1	0	1	1	1	1	0	0	1
**2**	E	**F14**	0	1	0	1	0	1	1	1	0	1
**3**	RP	**209**	0	0	0	0	1	1	1	0	1	0
**3**	RP	**210**	0	0	0	0	1	1	1	0	0	0
**3**	RP	**211**	1	0	0	0	1	1	1	0	0	0
**3**	RP	**212**	0	0	0	0	1	1	1	0	1	1
**3**	RP	**213**	0	0	0	0	1	1	1	1	1	1
**3**	RP	**214**	0	0	0	0	1	1	1	1	1	1
**3**	RP	**215**	0	0	0	0	1	1	1	1	1	1
**3**	RP	**216**	0	0	0	0	1	1	1	1	1	1
**3**	RP	**217**	0	0	0	0	1	1	1	1	1	1
**3**	RP	**218**	0	0	0	0	1	1	1	1	1	1
**3**	RP	**219**	0	0	0	0	1	1	1	1	1	1
**3**	RP	**220**	1	0	0	0	1	1	1	1	1	1
**3**	RP	**221**	0	0	0	0	1	1	1	1	1	1
**3**	RP	**222**	0	0	0	0	1	1	1	1	1	1
**3**	RP	**223**	0	0	0	0	1	1	1	1	1	1
**3**	RP	**224**	0	0	0	0	1	1	1	1	1	1
**3**	RP	**225**	0	0	1	0	1	1	1	1	1	1
**3**	RP	**226**	1	0	0	0	1	1	1	1	1	1
**3**	RP	**227**	1	0	0	0	1	1	1	1	1	1
**3**	RP	**228**	0	0	0	0	1	1	1	0	0	0
**3**	RP	**229**	1	0	0	0	0	1	1	0	1	1
**3**	R	**230**	0	0	0	0	1	1	1	0	1	1
**3**	R	**231**	0	0	0	0	1	1	1	0	0	0
**3**	R	**232**	0	0	0	0	1	1	1	1	0	0
**3**	R	**233**	0	0	1	0	1	1	1	0	0	0
**3**	R	**234**	0	0	0	0	1	1	1	1	0	0
**3**	R	**235**	0	0	0	0	1	1	1	0	0	0
**3**	R	**236**	1	0	0	0	1	1	1	1	1	1
**3**	R	**237**	0	0	0	0	1	1	1	1	1	1
**3**	R	**238**	0	0	0	0	1	1	1	0	1	1
**3**	R	**239**	0	0	0	0	1	1	1	0	1	1
**3**	R	**240**	0	0	0	0	1	1	1	0	1	1
**3**	R	**241**	0	0	1	0	1	1	1	1	1	1
**3**	R	**242**	0	0	0	0	1	1	1	1	1	1
**3**	R	**243**	0	0	0	0	1	1	1	1	0	0
**3**	R	**244**	0	0	0	0	1	1	1	0	0	0
**3**	R	**245**	0	0	0	0	1	1	1	0	0	0
**3**	R	**246**	0	0	0	0	0	1	1	0	0	0

Farm	Root compartment	Name ID	Gram	Fluorescence	PGP activity			Antagonistic activity				
					Siderophore production	Phosphate solubilization	Salt tolerance (NaCl 8%)	*Cmm*	*Pto*	*Pco*	*Xep*	*Fol*
**3**	R	**247**	0	0	0	0	0	1	1	0	1	1
**3**	R	**248**	0	0	0	0	0	1	1	1	1	1
**3**	R	**249**	0	0	1	0	1	1	1	1	0	0
**3**	R	**250**	0	0	0	0	1	1	1	0	0	0
**3**	E	**251**	0	0	0	0	1	1	1	1	0	0
**3**	E	**252**	0	0	0	0	0	1	1	1	1	1
**3**	E	**253**	0	0	0	0	1	1	1	1	1	1
**3**	E	**254**	0	0	0	0	1	1	1	0	0	0
**3**	E	**255**	0	0	0	0	1	1	1	1	1	1
**3**	E	**256**	0	0	0	0	0	1	1	0	0	0
**3**	E	**257**	0	0	0	1	1	1	1	1	1	1
**3**	E	**258**	0	0	0	0	1	1	1	1	1	1
**3**	E	**259**	0	0	0	1	1	1	1	1	0	1
**3**	E	**260**	0	0	0	1	1	1	1	0	1	1
**3**	E	**261**	0	0	0	1	1	1	1	1	1	1
**3**	E	**262**	0	0	0	1	1	1	1	1	1	1
**3**	E	**263**	0	0	0	1	1	1	1	1	1	1
**3**	E	**264**	0	0	0	1	1	1	1	1	1	1
**3**	E	**265**	0	0	0	0	1	1	1	1	1	1
**3**	E	**266**	1	0	0	1	1	1	1	1	1	1
**3**	E	**267**	0	0	0	1	1	1	1	1	1	1
**3**	E	**268**	0	0	0	1	1	1	1	1	1	1
**3**	E	**269**	0	0	0	1	1	1	1	0	1	1
**3**	E	**270**	0	0	0	1	1	1	1	1	1	1
**3**	E	**F19**	0	0	0	1	1	1	1	1	1	1
**3**	E	**F21**	0	0	0	1	1	1	1	1	1	1
**3**	E	**F22**	0	0	0	1	1	1	1	1	0	0
**3**	E	**F24**	0	0	0	1	1	1	1	0	0	0
**3**	R	**F32**	0	0	0	1	1	1	1	0	0	0
**3**	R	**F38**	0	0	0	1	1	1	1	1	1	1
**3**	R	**F39**	0	0	0	1	1	1	1	1	1	1
**3**	E	**F16**	0	1	0	1	1	1	1	1	1	1
**3**	E	**F17**	0	1	0	1	1	1	1	1	0	0
**3**	E	**F18**	0	1	0	1	1	1	1	0	0	0
**3**	E	**F20**	0	1	0	1	1	1	1	1	0	0
**3**	RP	**F25**	0	1	0	1	1	1	1	1	0	0
**3**	RP	**F26**	0	1	0	1	1	1	1	0	0	0
**3**	RP	**F27**	0	1	0	1	1	1	1	1	1	1
**3**	RP	**F28**	0	1	0	1	1	1	1	1	0	0
**3**	RP	**F29**	0	1	0	1	1	1	1	1	1	1
**3**	RP	**F30**	0	1	0	1	1	1	1	1	0	0
**3**	RP	**F31**	0	1	0	1	1	1	1	1	0	0
**3**	R	**F32**	0	1	0	1	1	1	1	1	1	1
**3**	R	**F33**	0	1	0	1	1	1	1	1	1	1
**3**	R	**F34**	0	1	0	1	1	1	1	1	0	0
**3**	R	**F35**	0	1	0	1	1	1	1	1	0	0
**3**	R	**F36**	0	1	0	1	1	1	1	1	1	1
**4**	R	**271**	0	0	1	0		1	1	1	1	1

Farm	Root compartment	Name ID	Gram	Fluorescence	PGP activity			Antagonistic activity				
					Siderophore production	Phosphate solubilization	Salt tolerance (NaCl 8%)	*Cmm*	*Pto*	*Pco*	*Xep*	*Fol*
**4**	R	**272**	0	0	0	0	1	1	1	1	1	1
**4**	R	**273**	0	0	1	0	1	0	0	0	0	1
**4**	R	**274**	1	0	1	0	1	1	1	1	0	1
**4**	R	**275**	0	0	1	0	1	1	1	1	1	1
**4**	R	**276**	0	0	0	0	1	1	1	1	1	1
**4**	R	**277**	0	0	1	0	1	0	0	0	0	1
**4**	R	**278**	0	0	1	0	1	0	0	0	0	1
**4**	R	**279**	0	0	0	0	1	0	0	0	0	1
**4**	R	**280**	0	0	0	0	1	1	1	1	1	1
**4**	R	**281**	1	0	1	0	1	0	0	1	1	1
**4**	R	**282**	0	0	1	0	1	1	1	1	1	1
**4**	R	**283**	1	0	0	0	1	0	0	0	0	1
**4**	R	**284**	1	0	1	0	1	0	0	0	0	1
**4**	R	**285**	1	0	1	0	1	0	0	0	0	1
**4**	R	**286**	1	0	1	0	1	1	0	1	1	1
**4**	R	**287**	0	0	0	0	1	0	1	0	0	1
**4**	R	**288**	0	0	0	0	1	0	0	0	0	1
**4**	R	**289**	0	0	0	0	1	1	1	1	1	1
**4**	R	**290**	1	0	0	0	1	1	1	1	1	1
**4**	R	**291**	1	0	1	0	1	0	1	0	0	1
**4**	RP	**292**	0	0	1	0	1	0	0	0	0	1
**4**	RP	**293**	0	0	1	0	1	0	0	0	0	1
**4**	RP	**294**	0	0	1	0	1	0	0	0	0	1
**4**	RP	**295**	0	0	0	0	1	0	0	0	0	1
**4**	RP	**296**	0	0	0	0	1	1	0	1	1	1
**4**	RP	**297**	0	0	0	0	1	0	1	0	0	1
**4**	RP	**298**	0	0	0	0	1	0	0	0	0	1
**4**	RP	**299**	0	0	1	0	1	0	0	0	0	1
**4**	RP	**300**	0	0	1	0	1	0	0	0	0	1
**4**	RP	**301**	1	0	0	0	1	0	0	0	0	1
**4**	RP	**302**	0	0	1	0	1	0	0	0	0	1
**4**	E	**303**	0	0	1	0	1	0	0	0	0	1
**4**	E	**304**	0	0	1	0	1	1	0	1	1	1
**4**	E	**305**	1	0	0	0	1	1	1	1	1	1
**4**	E	**306**	1	0	1	0	1	1	1	1	1	1
**4**	E	**307**	0	0	0	0	1	0	1	0	0	1
**4**	E	**308**	0	0	0	0	1	0	0	0	0	1
**4**	E	**309**	0	0	1	0	1	0	0	0	0	1
**4**	E	**310**	0	0	1	0	1	1	0	1	1	1
**4**	R	**311**	0	0	1	0	1	1	1	1	1	1
**4**	R	**312**	1	0	1	0	1	1	1	1	1	1
**4**	R	**313**	0	0	0	0	1	1	1	1	1	1
**4**	R	**314**	1	0	1	0	1	0	1	0	0	1
**4**	R	**315**	0	0	0	0	1	1	1	1	1	1
**4**	R	**316**	0	0	0	0	1	1	1	1	1	1
**4**	R	**317**	1	0	0	0	1	1	1	1	1	1
**4**	R	**318**	0	0	1	0	1	0	1	1	0	1
**4**	R	**319**	0	0	1	1	1	1	1	1	1	1
**4**	R	**320**	1	0	1	1	1	1	1	1	1	1

**Farm**	**Root compartment**	**Name ID**	**Gram**	**Fluorescence**	**PGP activity**			**Antagonistic activity**				
					**Siderophore production**	**Phosphate solubilization**	**Salt tolerance (NaCl 8%)**	***Cmm***	***Pto***	***Pco***	***Xep***	***Fol***
**4**	R	**321**	1	0	1	1	1	1	1	1	1	1
**4**	R	**322**	1	0	1	1	1	1	1	1	1	1
**4**	R	**323**	0	0	1	1	1	1	0	0	0	1
**4**	R	**324**	1	0	1	1	1	0	1	1	1	1
**4**	R	**325**	1	0	1	1	1	1	0	0	1	1
**4**	R	**326**	1	0	1	1	1	1	0	0	1	1
**4**	R	**327**	1	0	1	1	1	0	1	1	1	1
**4**	R	**328**	0	0	1	1	1	0	1	1	1	1
**4**	R	**329**	0	0	1	1	1	1	1	1	1	1
**4**	R	**330**	1	0	1	1	1	1	1	1	1	1
**4**	RP	**331**	0	0	1	1	1	1	0	0	0	1
**4**	RP	**332**	0	0	1	1	1	1	0	0	1	1
**4**	RP	**333**	0	0	1	1	1	1	0	0	1	1
**4**	RP	**334**	1	0	1	1	1	1	1	1	1	1
**4**	RP	**335**	0	0	1	1	1	1	0	0	0	1
**4**	RP	**336**	0	0	1	1	1	1	1	1	0	1
**4**	RP	**337**	0	0	1	1	1	1	0	0	0	1
**4**	E	**338**	0	0	1	1	1	1	1	1	1	1
**4**	E	**339**	0	0	1	1	1	1	0	0	0	1
**4**	E	**340**	1	0	1	1	1	1	0	0	1	1
**4**	E	**341**	0	0	1	1	1	1	0	0	1	1
**4**	E	**342**	0	0	1	1	1	1	1	1	1	1
**4**	E	**343**	0	0	1	1	1	1	1	1	1	1
**4**	E	**344**	1	0	1	1	1	0	1	1		1
**4**	E	**345**	0	0	1	1	1	1	1	1	0	1
**4**	E	**346**	0	0	1	1	1	1	0	0	1	1
**4**	E	**347**	1	0	1	1	1	1	1	0	1	1
**4**	E	**348**	0	0	1	1	1	1	0	0	0	1
**4**	E	**349**	1	0	1	1	1	1	0	0	1	1
**4**	E	**350**	0	0	1	1	1	1	0	0	1	1
**4**	E	**351**	1	0	1	1	1	1	1	1	1	1
**4**	E	**352**	1	0	1	1	1	1	1	1	0	1
**4**	E	**353**	0	0	1	1	1	1	1	1	0	1
**4**	E	**354**	0	0	1	1	1	1	0	0	0	1
**4**	RP	**355**	0	0	1	1	1	1	1	0	1	1
**4**	RP	**356**	0	0	1	1	1	1	1	1	1	1
**4**	RP	**357**	0	0	1	1	1	1	1	1	1	1
**4**	RP	**358**	0	0	1	1	1	1	1	1	1	1
**4**	R	**359**	0	0	1	1	1	1	1	1	0	1
**4**	R	**360**	0	0	1	1	1	1	1	1	0	1
**4**	R	**361**	0	0	1	1	1	1	1	1	1	1
**4**	R	**362**	1	0	1	1	1	1	0	1	0	1
**4**	R	**363**	0	0	1	1	1	1	1	1	1	1
**4**	R	**364**	1	0	1	1	1	1	1	1	1	1
**4**	R	**365**	0	0	1	1	1	1	1	1	1	1
**4**	R	**366**	0	0	1	1	1	1	1	1	1	1
**4**	R	**367**	0	0	1	0	1	1	0	0	0	1
**4**	E	**368**	0	0	1	0	1	1	1	1	1	1

Farm	Root compartment	Name ID	Gram	Fluorescence	PGP activity			Antagonistic activity				
					Siderophore production	Phosphate solubilization	Salt tolerance (NaCl 8%)	*Cmm*	*Pto*	*Pco*	*Xep*	*Fol*
**4**	E	**369**	1	0	1	0	1	0	0	0	1	1
**4**	E	**370**	1	0	0	1	1	1	1	1	1	1
**4**	E	**371**	1	0	1	1	1	1	1	1	1	1
**4**	E	**372**	0	0	1	1	1	1	1	1	1	1
**4**	E	**373**	0	0	1	1	1	1	1	1	1	1
**4**	RP	**374**	0	0	0	0	1	1	1	1	1	1
**4**	RP	**375**	0	0	1	0	1	1	1	1	1	1
**4**	RP	**376**	0	0	1	0	1	1	1	1	1	1
**4**	RP	**377**	0	0	1	0	1	0	0	0	0	1
**4**	RP	**378**	0	0	1	1	1	1	1	0	1	1
**4**	RP	**379**	1	0	1	1	1	1	1	1	1	1
**4**	RP	**380**	0	0	0	1	1	1	1	1	1	1
**4**	RP	**381**	0	0	1	0	1	1	0	1	1	1
**4**	RP	**382**	0	0	1	0	0	0	0	0	0	1
**4**	RP	**F37**	0	0	1	0	1	1	0	1	0	1
**4**	RP	**F38**	0	0	1	0	1	1	0	0	0	1
**4**	RP	**F39**	0	0	1	0	1	1	0	0	0	1
**4**	RP	**F40**	0	0	1	0	1	1	0	0	0	1
**4**	RP	**F41**	0	1	1	0	0	1	0	0	0	1
**4**	E	**F42**	0	1	1	0	0	1	0	0	0	1
**4**	RP	**F43**	0	0	1	0	0	1	0	0	0	1
**4**	RP	**F44**	0	0	1	0	1	1	0	0	0	1
**4**	RP	**F45**	0	0	1	0	0	1	0	0	0	1
**4**	E	**F46**	0	1	1	0	1	1	0	0	0	1
**4**	RP	**F47**	0	0	1	0	1	1	0	0	0	1
**4**	E	**F48**	0	1	1	0	0	1	0	0	0	1
**4**	RP	**F49**	0	0	1	0	0	1	0	0	0	1
**4**	E	**F50**	0	1	1	0	0	1	0	0	0	1
**4**	RP	**F51**	0	0	1	0	0	1	0	0	0	1
**4**	E	**F52**	0	1	1	0	0	1	0	0	0	1
**4**	E	**F53**	0	1	1	0	0	1	0	0	0	1
**4**	E	**F54**	0	1	0	0	1	1	0	0	0	1
**4**	R	**F55**	0	1	1	1	1	0	0	0	0	1
**4**	E	**F56**	0	1	1	0	1	1	1	1	1	1
**4**	R	**F57**	0	1	1	0	1	1	0	0	0	1
**4**	E	**F58**	0	1	1	0	0	1	0	0	0	1
**4**	R	**F59**	0	1	1	0	1	1	0	0	0	1
**4**	E	**F60**	0	1	1	0	1	1	1	1	1	1

Table S2: Principal Component Analysis (PCA) table of the full dataset on 424 bacteria, showing relationships between phenotypic traits (Gram reaction, fluorescence production, siderophore production, phosphate solubilisation, salt tolerance, antagonist activity against *Cmm*, *Pco*, *Pto*, *Xep*, and *Fol*) and bacterial isolates.

Bacteria ID	PC1	PC2
**1**	-0.272	-0.295
**2**	-0.199	-0.4
**3**	-0.709	-0.643
**4**	-0.659	-0.708
**5**	-0.659	-0.708
**6**	-0.659	-0.708
**7**	-0.199	-0.4
**8**	-0.272	-0.295
**9**	-0.659	-0.708
**10**	-0.384	0.08
**11**	0.076	0.388
**12**	-0.384	0.08
**13**	0.076	0.388
**14**	-0.92	0.359
**15**	-0.92	0.359
**16**	-0.734	-0.121
**17**	-0.92	0.359
**18**	-0.459	0.667
**19**	-0.459	0.667
**20**	0.076	0.388
**21**	0.076	0.388
**22**	0.076	0.388
**23**	0.076	0.388
**24**	0.076	0.388
**25**	-0.456	-0.285
**26**	-1.451	-0.313
**27**	0.076	0.388
**28**	0.026	0.452
**29**	0.076	0.388
**30**	0.076	0.388
**31**	-1.144	0.255
**32**	-1.144	0.255
**33**	-0.92	0.359
**34**	-0.92	0.359
**35**	-0.97	0.424
**36**	0.076	0.388
**37**	0.026	0.452
**38**	-0.92	0.359
**39**	0.076	0.388
**40**	-0.149	0.284
**41**	0.076	0.388
**42**	-0.199	-0.4

Bacteria ID	PC1	PC2
**43**	-0.199	-0.4
**44**	-0.199	-0.4
**45**	0.076	0.388
**46**	0.076	0.388
**47**	-0.199	-0.4
**48**	-0.199	-0.4
**49**	-0.072	-0.033
**50**	0.389	0.275
**51**	-0.072	-0.033
**52**	0.114	-0.513
**53**	0.114	-0.513
**54**	0.114	-0.513
**55**	0.114	-0.513
**56**	0.624	-0.846
**57**	0.389	0.275
**58**	0.389	0.275
**59**	0.316	0.379
**60**	0.316	0.379
**61**	0.389	0.275
**62**	0.316	0.379
**63**	0.316	0.379
**64**	0.041	-0.409
**65**	-0.807	-0.016
**66**	-0.532	0.771
**67**	-0.719	-0.233
**68**	-0.444	0.554
**69**	-0.219	0.658
**70**	-0.444	0.554
**91**	-0.146	0.554
**92**	0.899	-0.058
**93**	0.586	0.055
**94**	0.142	0.237
**95**	0.586	0.055
**96**	0.364	0.221
**97**	0.917	-0.051
**98**	0.826	0.046
**99**	0.99	-0.155
**100**	0.076	0.388
**101**	0.003	0.492
**102**	-0.022	0.438
**103**	0.076	0.388
**104**	0.586	0.055
**105**	0.899	-0.058
**106**	-0.532	0.771
**107**	0.389	0.275
**108**	0.003	0.492
**109**	-0.532	0.771

Bacteria ID	PC1	PC2
**110**	-0.459	0.667
**111**	0.076	0.388
**112**	-0.368	0.57
**113**	0.076	0.388
**114**	0.076	0.388
**115**	0.364	0.221
**116**	0.586	0.055
**117**	0.586	0.055
**118**	0.003	0.492
**119**	0.076	0.388
**120**	0.076	0.388
**121**	-0.401	0.789
**122**	-0.179	0.773
**123**	0.826	0.046
**124**	0.586	0.055
**125**	0.586	0.055
**126**	0.076	0.388
**127**	0.514	0.159
**128**	-0.532	0.771
**129**	-0.491	0.886
**130**	-0.401	0.789
**131**	0.826	0.046
**132**	0.586	0.055
**133**	-0.459	0.667
**134**	0.586	0.055
**135**	0.604	0.063
**136**	0.167	0.291
**137**	0.167	0.291
**138**	0.899	-0.058
**139**	0.677	-0.042
**140**	0.586	0.055
**141**	0.48	0.178
**142**	-0.219	0.658
**143**	0.48	0.178
**144**	0.481	0.378
**145**	-0.441	0.675
**146**	0.003	0.492
**147**	0.389	0.275
**148**	0.48	0.178
**149**	0.389	0.275
**150**	0.48	0.178
**151**	0.917	-0.051
**152**	0.167	0.291
**153**	-0.368	0.57
**154**	0.677	-0.042
**155**	0.899	-0.058

Bacteria ID	PC1	PC2
**156**	-0.368	0.57
**157**	0.677	-0.042
**158**	0.142	0.237
**159**	-0.368	0.57
**160**	0.167	0.291
**161**	0.094	0.396
**162**	0.677	-0.042
**163**	0.48	0.178
**164**	-0.056	0.457
**165**	0.586	0.055
**166**	0.677	-0.042
**167**	0.167	0.291
**168**	-0.459	0.667
**169**	0.076	0.388
**170**	-0.368	0.57
**171**	0.48	0.178
**172**	0.48	0.178
**173**	-0.368	0.57
**174**	-0.056	0.457
**175**	0.48	0.178
**176**	-0.146	0.554
**177**	-0.056	0.457
**178**	-0.056	0.457
**179**	0.99	-0.155
**180**	0.389	0.275
**181**	-0.459	0.667
**182**	0.364	0.221
**183**	0.389	0.275
**184**	0.389	0.275
**185**	0.899	-0.058
**186**	0.899	-0.058
**187**	-0.088	0.676
**188**	0.389	0.275
**189**	0.389	0.275
**190**	0.389	0.275
**191**	0.899	-0.058
**192**	0.48	0.178
**193**	0.389	0.275
**194**	-0.056	0.457
**195**	-0.146	0.554
**196**	0.389	0.275
**197**	-0.146	0.554
**198**	-0.146	0.554
**199**	-0.146	0.554
**200**	0.389	0.275
**201**	-0.056	0.457
**202**	0.389	0.275

Bacteria ID	PC1	PC2
**203**	0.958	0.064
**204**	0.867	0.16
**205**	0.899	-0.058
**206**	-0.146	0.554
**207**	-0.129	0.562
**208**	0.826	0.046
**F11**	-0.146	0.554
**F13**	0.389	0.275
**F1**	0.314	0.285
**F2**	0.339	0.339
**F3**	-0.269	0.723
**F4**	-0.196	0.619
**F5**	-0.196	0.619
**F6**	-0.196	0.619
**F7**	-0.146	0.554
**F8**	-0.146	0.554
**F9**	0.266	0.444
**F10**	0.266	0.444
**F12**	-0.196	0.619
**F14**	0.241	0.39
**209**	0.044	0.607
**210**	-0.491	0.886
**211**	-0.401	0.789
**212**	0.076	0.388
**213**	0.586	0.055
**214**	0.586	0.055
**215**	0.586	0.055
**216**	0.586	0.055
**217**	0.586	0.055
**218**	0.586	0.055
**219**	0.586	0.055
**220**	0.677	-0.042
**221**	0.586	0.055
**222**	0.586	0.055
**223**	0.586	0.055
**224**	0.586	0.055
**225**	0.312	-0.733
**226**	0.677	-0.042
**227**	0.677	-0.042
**228**	-0.491	0.886
**229**	0.094	0.396
**230**	0.076	0.388
**231**	-0.491	0.886
**232**	0.019	0.553
**233**	-0.766	0.098
**234**	0.019	0.553

Bacteria ID	PC1	PC2
**235**	-0.491	0.886
**236**	0.677	-0.042
**237**	0.586	0.055
**238**	0.076	0.388
**239**	0.076	0.388
**240**	0.076	0.388
**241**	0.312	-0.733
**242**	0.586	0.055
**243**	0.019	0.553
**244**	-0.491	0.886
**245**	-0.491	0.886
**246**	-0.564	0.99
**247**	0.003	0.492
**248**	0.514	0.159
**249**	-0.256	-0.235
**250**	-0.491	0.886
**251**	0.019	0.553
**252**	0.514	0.159
**253**	0.586	0.055
**254**	-0.491	0.886
**255**	0.586	0.055
**256**	-0.564	0.99
**257**	0.899	-0.058
**258**	0.586	0.055
**259**	0.364	0.221
**260**	0.389	0.275
**261**	0.899	-0.058
**262**	0.899	-0.058
**263**	0.899	-0.058
**264**	0.899	-0.058
**265**	0.586	0.055
**266**	0.99	-0.155
**267**	0.899	-0.058
**268**	0.899	-0.058
**269**	0.389	0.275
**270**	0.899	-0.058
**F19**	0.899	-0.058
**F21**	0.899	-0.058
**F22**	0.332	0.44
**F24**	-0.179	0.773
**F32**	-0.179	0.773
**F38**	0.899	-0.058
**F39**	0.899	-0.058
**F16**	0.849	0.006
**F17**	0.282	0.504
**F18**	-0.229	0.837
**F20**	0.282	0.504

Bacteria ID	PC1	PC2
**F25**	0.282	0.504
**F26**	-0.229	0.837
**F27**	0.849	0.006
**F28**	0.282	0.504
**F29**	0.849	0.006
**F30**	0.282	0.504
**F31**	0.282	0.504
**F32**	0.849	0.006
**F33**	0.849	0.006
**F34**	0.282	0.504
**F35**	0.282	0.504
**F36**	0.849	0.006
**271**	0.239	-0.628
**272**	0.586	0.055
**273**	-1.419	-0.532
**274**	-0.133	-0.55
**275**	0.312	-0.733
**276**	0.586	0.055
**277**	-1.419	-0.532
**278**	-1.419	-0.532
**279**	-1.144	0.255
**280**	0.586	0.055
**281**	-0.283	-1.241
**282**	0.312	-0.733
**283**	-1.054	0.159
**284**	-1.328	-0.629
**285**	-1.328	-0.629
**286**	-0.058	-1.137
**287**	-0.684	0.563
**288**	-1.144	0.255
**289**	0.586	0.055
**290**	0.677	-0.042
**291**	-0.868	-0.321
**292**	-1.419	-0.532
**293**	-1.419	-0.532
**294**	-1.419	-0.532
**295**	-1.144	0.255
**296**	0.126	-0.253
**297**	-0.684	0.563
**298**	-1.144	0.255
**299**	-1.419	-0.532
**300**	-1.419	-0.532
**301**	-1.054	0.159
**302**	-1.419	-0.532
**303**	-1.419	-0.532
**304**	-0.149	-1.041

Bacteria ID	PC1	PC2
**305**	0.677	-0.042
**306**	0.402	-0.829
**307**	-0.684	0.563
**308**	-1.144	0.255
**309**	-1.419	-0.532
**310**	-0.149	-1.041
**311**	0.312	-0.733
**312**	0.402	-0.829
**313**	0.586	0.055
**314**	-0.868	-0.321
**315**	0.586	0.055
**316**	0.586	0.055
**317**	0.677	-0.042
**318**	-0.448	-0.557
**319**	0.624	-0.846
**320**	0.715	-0.943
**321**	0.715	-0.943
**322**	0.715	-0.943
**323**	-0.882	-0.542
**324**	0.491	-1.046
**325**	-0.256	-0.917
**326**	-0.256	-0.917
**327**	0.491	-1.046
**328**	0.4	-0.95
**329**	0.624	-0.846
**330**	0.715	-0.943
**331**	-0.882	-0.542
**332**	-0.346	-0.821
**333**	-0.346	-0.821
**334**	0.715	-0.943
**335**	-0.882	-0.542
**336**	0.089	-0.567
**337**	-0.882	-0.542
**338**	0.624	-0.846
**339**	-0.882	-0.542
**340**	-0.256	-0.917
**341**	-0.346	-0.821
**342**	0.624	-0.846
**343**	0.624	-0.846
**344**	-0.045	-0.767
**345**	0.089	-0.567
**346**	-0.346	-0.821
**347**	0.205	-0.61
**348**	-0.882	-0.542
**349**	-0.256	-0.917
**350**	-0.346	-0.821
**351**	0.715	-0.943

Bacteria ID	PC1	PC2
**352**	0.18	-0.663
**353**	0.089	-0.567
**354**	-0.882	-0.542
**355**	0.114	-0.513
**356**	0.624	-0.846
**357**	0.624	-0.846
**358**	0.624	-0.846
**359**	0.089	-0.567
**360**	0.089	-0.567
**361**	0.624	-0.846
**362**	-0.281	-0.971
**363**	0.624	-0.846
**364**	0.715	-0.943
**365**	0.624	-0.846
**366**	0.624	-0.846
**367**	-1.194	-0.428
**368**	0.312	-0.733
**369**	-0.793	-0.908
**370**	0.99	-0.155
**371**	0.715	-0.943
**372**	0.624	-0.846
**373**	0.624	-0.846
**374**	0.586	0.055
**375**	0.312	-0.733
**376**	0.312	-0.733
**377**	-1.419	-0.532
**378**	0.114	-0.513
**379**	0.715	-0.943
**380**	0.899	-0.058
**381**	-0.149	-1.041
**382**	-1.492	-0.428
**F37**	-0.684	-0.762
**F38**	-1.194	-0.428
**F39**	-1.194	-0.428
**F40**	-1.194	-0.428
**F41**	-1.317	-0.259
**F42**	-1.317	-0.259
**F43**	-1.267	-0.324
**F44**	-1.194	-0.428
**F45**	-1.267	-0.324
**F46**	-1.244	-0.364
**F47**	-1.194	-0.428
**F48**	-1.317	-0.259
**F49**	-1.267	-0.324
**F50**	-1.317	-0.259
**F51**	-1.267	-0.324

Bacteria ID	PC1	PC2
**F52**	-1.317	-0.259
**F53**	-1.317	-0.259
**F54**	-0.97	0.424
**F55**	-1.156	-0.581
**F56**	0.262	-0.668
**F57**	-1.244	-0.364
**F58**	-1.317	-0.259
**F59**	-1.244	-0.364
**F60**	0.262	-0.668

Table S3: Molecular identification of the endophytic bacteria based on the 16S rRNA gene sequence.

Strain ID	Best hit (ref_seq)						This study GenBank accession No.
	Order	Family	Genus	Species	Ident. %	NCBI Acc. No.	
**124**	Pseudomonadales	Moraxellaceae	*Acinetobacter*	*Acinetobacter baumannii*	100	CP050388.1	MW130753
**170**	Pseudomonadales	Moraxellaceae	*Acinetobacter*	*Acinetobacter baumannii*	100	CP050388.1	MW130754
**123**	Pseudomonadales	Moraxellaceae	*Acinetobacter*	*Acinetobacter baumannii*	100	MT256198.1	MW130755
**307**	Pseudomonadales	Moraxellaceae	*Acinetobacter*	*Acinetobacter baumannii*	100	MT256198.1	MW130756
**263**	Bacillales	Bacillaceae	*Bacillus*	*Bacillus velezensis*	100	MN559711.1	MW130757
**373**	Bacillales	Bacillaceae	*Bacillus*	*Bacillus subtilis*	100	CP051860.1	MW130758
**306**	Bacillales	Bacillaceae	*Bacillus*	*Bacillus velezensis*	100	CP051463.1	MW130759
**341**	Bacillales	Bacillaceae	*Bacillus*	*Bacillus velezensis*	100	KY927398.1	MW130760
**268**	Bacillales	Bacillaceae	*Bacillus*	*Bacillus megaterium*	99	KT883839.1	MW130761
**261**	Bacillales	Bacillaceae	*Bacillus*	*Bacillus velezensis*	99	CP051463.1	MW130762
**304**	Bacillales	Bacillaceae	*Bacillus*	*Bacillus velezensis*	100	CP051463.1	MW130763
**305**	Bacillales	Bacillaceae	*Bacillus*	*Bacillus velezensis*	100	CP051463.1	MW130764
**255**	Bacillales	Bacillaceae	*Bacillus*	*Bacillus velezensis*	100	MT365117.1	MW130765
**265**	Bacillales	Bacillaceae	*Bacillus*	*Bacillus velezensis*	100	MT365117.1	MW130766
**351**	Bacillales	Bacillaceae	*Bacillus*	*Bacillus amyloliquefaciens*	100	MK501609.1	MW130767
**347**	Bacillales	Bacillaceae	*Bacillus*	*Bacillus amyloliquefaciens*	100	MK501609.1	MW130768
**374**	Bacillales	Bacillaceae	*Bacillus*	*Bacillus velezensis*	100	KY927398.1	MW130769
**257**	Bacillales	Bacillaceae	*Bacillus*	*Bacillus amiloliquefaciens*	100	MK501609.1	MW130770
**136**	Bacillales	Bacillaceae	*Bacillus*	*Bacillus velezensis*	99	MN654121.1	MW130771
**270**	Bacillales	Bacillaceae	*Bacillus*	*Bacillus amiloliquefaciens*	100	MK501609.1	MW130772
**372**	Bacillales	Bacillaceae	*Bacillus*	*Bacillus subtilis*	100	MT081484.1	MW130773
**260**	Bacillales	Bacillaceae	*Bacillus*	*Bacillus subtilis*	100	KU729674.1	MW130774
**338**	Bacillales	Bacillaceae	*Bacillus*	*Bacillus sp.*	100	CP040881.1	MW130775
**370**	Bacillales	Bacillaceae	*Bacillus*	*Bacillus sp.*	100	CP040881.1	MW130776
**269**	Bacillales	Bacillaceae	*Bacillus*	*Bacilus velenzensis*	100	CP024922.1	MW130777
**132**	Enterobacteriales	Enterobacteriaceae	*Lelliottia*	*Lelliottia sp.*	97	JN853247.1	MW130778
**127**	Enterobacteriales	Enterobacteriaceae	*Lelliottia*	*Lelliottia sp.*	97	JN853247.1	MW130779
**198**	Enterobacteriales	Enterobacteriaceae	*Lelliottia*	*Lelliottia sp.*	97	JN853247.1	MW130780
**135**	Enterobacteriales	Enterobacteriaceae	*Lelliottia*	*Lelliottia sp.*	97	JN853247.1	MW130781
**267**	Enterobacteriales	Enterobacteriaceae	*Enterobacter*	*Enterobacter cancerogenus*	97	FJ976582.1	MW130782
**f20**	Enterobacteriales	Enterobacteriaceae	*Enterobacter*	*Enterobacter cancerogenus*	97	FJ976582.1	MW130783
**f21**	Enterobacteriales	Enterobacteriaceae	*Enterobacter*	*Enterobacter cancerogenus*	97	FJ976582.1	MW130784
**f18**	Enterobacteriales	Enterobacteriaceae	*Enterobacter*	*Enterobacter cancerogenus*	97	FJ976582.1	MW130785
**252**	Enterobacteriales	Enterobacteriaceae	*Enterobacter*	*Enterobacter cancerogenus*	97	FJ976582.1	MW130786
**f17**	Enterobacteriales	Enterobacteriaceae	*Enterobacter*	*Enterobacter mori*	97	KJ589489.1	MW130787
**f19**	Enterobacteriales	Enterobacteriaceae	*Enterobacter*	*Enterobacter cancerogenus*	97	FJ976582.1	MW130788
**254**	Enterobacteriales	Enterobacteriaceae	*Enterobacter*	*Enterobacter cancerogenus*	97	FJ976582.1	MW130789

	Best hit (ref_seq)						This study GenBank accession No.
**Strain ID**	**Order**	**Family**	**Genus**	**Species**	**Ident. %**	**NCBI Acc. No.**	
**266**	Enterobacteriales	Enterobacteriaceae	*Enterobacter*	*Enterobacter cancerogenus*	97	FJ976582.1	MW130790
**128**	Enterobacteriales	Enterobacteriaceae	*Lelliottia*	*Lelliottia sp.*	97	JN853247.1	MW130791
**f52**	Enterobacteriales	Enterobacteriaceae	*Enterobacter*	*Enterobacter tabaci*	97	MF682952.1	MW130792
**126**	Enterobacteriales	Enterobacteriaceae	*Lelliottia*	*Lelliottia sp.*	97	JN853247.1	MW130793
**200**	Enterobacteriales	Enterobacteriaceae	*Lelliottia*	*Lelliottia sp.*	96	JN853247.1	MW130794
**202**	Enterobacteriales	Enterobacteriaceae	*Lelliottia*	*Lelliottia sp.*	97	JN853247.1	MW130795
**259**	Enterobacteriales	Enterobacteriaceae	*Lelliottia*	*Lelliottia sp.*	97	JN853247.1	MW130796
**176**	Enterobacteriales	Enterobacteriaceae	*Lelliottia*	*Lelliottia sp.*	97	JN853247.1	MW130797
**310**	Enterobacteriales	Enterobacteriaceae	*Enterobacter*	*Enterobacter cancerogenus*	97	FJ976582.1	MW130798
**49**	Enterobacteriales	Enterobacteriaceae	*Ewingella*	*Ewingella americana*	100	MT101745.1	MW130799
**48**	Enterobacteriales	Enterobacteriaceae	*Ewingella*	*Ewingella americana*	99	MT101745.1	MW130800
**35**	Enterobacteriales	Enterobacteriaceae	*Ewingella*	*Ewingella americana*	99	KY126991.1	MW130801
**346**	Enterobacteriales	Enterobacteriaceae	*Pantoea*	*Pantoea sp.*	97	MK229045.1	MW130802
**353**	Enterobacteriales	Enterobacteriaceae	*Pantoea*	*Pantoea sp.*	97	MH884045.1	MW130803
**264**	Enterobacteriales	Enterobacteriaceae	*Enterobacter*	*Enterobacter cancerogenus*	97	FJ976582.1	MW130804
**344**	Enterobacteriales	Enterobacteriaceae	*Pantoea*	*Pantoea sp.*	97	MK229045.1	MW130805
**345**	Enterobacteriales	Enterobacteriaceae	*Pantoea*	*Pantoea sp.*	97	MK229045.1	MW130806
**342**	Enterobacteriales	Morganellaceaea	*Providencia*	*Providencia vermicola*	99	KX394623.1	MW130807
**350**	Enterobacteriales	Morganellaceaea	*Providencia*	*Providencia vermicola*	99	MK942706.1	MW130808
**349**	Enterobacteriales	Morganellaceaea	*Providencia*	*Providencia vermicola*	99	MK942706.1	MW130809
**f1**	Pseudomonadales	Pseudomonadaceae	*Pseudomonas*	*Pseudomonas citronellolis*	100	KM210226.1	MW130810
**172**	Pseudomonadales	Pseudomonadaceae	*Pseudomonas*	*Pseudomonas plecoglossicida*	100	MT367715.1	MW130811
**f14**	Pseudomonadales	Pseudomonadaceae	*Pseudomonas*	*Pseudomonas putida*	100	LN866622.1	MW130812
**f53**	Pseudomonadales	Pseudomonadaceae	*Pseudomonas*	*Pseudomonas monteilii*	100	MH603875.1	MW130813
**125**	Pseudomonadales	Pseudomonadaceae	*Pseudomonas*	*Pseudomonas plecoglossicida*	100	MT367715.1	MW130814
**201**	Pseudomonadales	Pseudomonadaceae	*Pseudomonas*	*Pseudomonas plecoglossicida*	100	MT367715.1	MW130815
**168**	Pseudomonadales	Pseudomonadaceae	*Pseudomonas*	*Pseudomonas plecoglossicida*	100	KJ819579.1	MW130816
**169**	Pseudomonadales	Pseudomonadaceae	*Pseudomonas*	*Pseudomonas plecoglossicida*	100	MT367715.1	MW130817
**174**	Pseudomonadales	Pseudomonadaceae	*Pseudomonas*	*Pseudomonas plecoglossicida*	100	MT367715.1	MW130818
**352**	Pseudomonadales	Pseudomonadaceae	*Pseudomonas*	*Pseudomonas plecoglossicida*	100	MT367715.1	MW130819
**173**	Pseudomonadales	Pseudomonadaceae	*Pseudomonas*	*Pseudomonas plecoglossicida*	100	MT367715.1	MW130820
**f2**	Pseudomonadales	Pseudomonadaceae	*Pseudomonas*	*Pseudomonas plecoglossicida*	100	MT367715.1	MW130821
**f10**	Pseudomonadales	Pseudomonadaceae	*Pseudomonas*	*Pseudomonas plecoglossicida*	100	MT367715.1	MW130822
**f56**	Pseudomonadales	Pseudomonadaceae	*Pseudomonas*	*Pseudomonas plecoglossicida*	100	MT367715.1	MW130823
**f60**	Pseudomonadales	Pseudomonadaceae	*Pseudomonas*	*Pseudomonas plecoglossicida*	100	MT367715.1	MW130824
**f42**	Pseudomonadales	Pseudomonadaceae	*Pseudomonas*	*Pseudomonas putida*	100	CP026115.2	MW130825
**f48**	Pseudomonadales	Pseudomonadaceae	*Pseudomonas*	*Pseudomonas putida*	100	CP026115.2	MW130826

	Best hit (ref_seq)						This study GenBank accession No.
**Strain ID**	**Order**	**Family**	**Genus**	**Species**	**Ident. %**	**NCBI Acc. No.**	
**171**	Pseudomonadales	Pseudomonadaceae	*Pseudomonas*	*Pseudomonas plecoglossicida*	100	MT367715.1	MW130827
**f9**	Pseudomonadales	Pseudomonadaceae	*Pseudomonas*	*Pseudomonas plecoglossicida*	100	MT367715.1	MW130828
**f46**	Pseudomonadales	Pseudomonadaceae	*Pseudomonas*	*Pseudomonas putida*	100	LN866622.1	MW130829

Table S4: Accession numbers of the sequences of the type strains used as references in the dendrogram in Figure 6 in this study.

Species	Type Strain	Sequence accession no. (16S rRNA gene)
Bacillus subtilis	ATCC 6051^T	AJ276351
B. pumilus	ATCC 7061^T	AY876289
B. megaterium	ATCC 14581^T	D16273
B. siamensis	KCTC 13613^T	GQ281299
B. mojavensis	ATCC 51516^T	AB021191
B. amyloliquefaciens	ATCC 23350^T	AB006920
B. velezensis	CCUG 50740^T	AY603658
B. thuringiensis	ATCC 10792^T	D16281
B. cereus	ATCC 14579^T	AE016877
Acinetobacter baumannii	DSM 30007^T	NR_117677
Pseudomonas citronellolis	ATCC 13674^T	Z76659
P. plecoglossicida	ATCC 700383^T	AB009457
P. putida	ATCC 12633^T	D84020
P. monteilii	ATCC 700476^T	AF064458
P. mosselii	CIP 105259^T	AF072688
P. entomophila	CCUG 61470^T	AY907566
P. parafulva	DSM 17004^T	AB060132
P. fulva	DSM 17717^T	AB060136
P. syingae pv. syingae	NCPPB 281^T	DQ318866
P. fluorescens	DSM 50090^T	D84013
P. aeruginosa	DSM 50071^T	HE978771
Enterobacter cancerogenus	LMG 2693^T	NR 116756.1
E. mori	LMG 25706^T	NZ_GL890774
E. tabaci	KACC 17832^T	NR_146667
Ewingella americana	ATCC 33852^T	JMPJ01000013
Providencia vermicola	DSM 17385^T	NR_042415
Lelliottia amnigena	ATCC 33731^T	AB004749
L. nimipressuralis	DSM 18955^T	KF516260
Pantoea agglomerans	DSM 3493^T	NR_041978

Table S5: Effect of the treatments by soil drenching of tomato plantlets grown in pots using bacterial endophytes belonging to the genus *Pseudomonas* and *Bacillus*. Plant height, fresh and dry weight of roots and shoots, dry matter and root/shoot ratio was measured 30 days after the treatment.

Bacterial strains	Plant height		Root			Shoot			R/S
	T30	Gain (%)	fw	dw	DM	fw	dw	DM	
P. plecoglossicida _171	29.33±1.15 abc	11.36	0.70 ab	0.57 a	83.41 c	4.20 a	1.84 a	44.06 a	0.31 ab
P. plecoglossicida _172	29.83±1.89 abc	12.85	0.90 ab	0.53 a	60.14 ab	5.10 a	2.23 b	43.71 a	0.24 ab
*P. citronellolis*_f1	31.33±3.06 bc	17.02	1.13 b	0.63 a	56.00 a	5.13 a	2.23 b	44.62 a	0.29 ab
*P. monteilii*_f53	29.00±1.00 abc	10.4	0.93 ab	0.59 a	62.81 ab	4.63 a	1.73 a	37.82 a	0.34 b
*P. plecoglossicida*_f56	32.33±1.15 bc	19.59	0.66 a	0.58 a	87.22 c	5.36 a	2.26 b	42.41 a	0.25 ab
B. velezensis _261	31.67±2.52 bc	17.89	1.08 ab	0.54 a	54.42 a	5.03 a	2.22 b	46.18 a	0.24 ab
*B. velezensis*_263	30.33±0.58 abc	14.29	0.63 a	0.49 a	76.98 bc	4.90 a	2.19 b	44.76 a	0.22 a
B. velezensis _265	27.67±1.53 ab	6.02	1.13 b	0.65 a	57.77 ab	5.03 a	1.91 ab	37.98 a	0.35 b
*B. megaterium*_268	26.33±1.53 a	1.27	0.90 ab	0.62 a	70.00 abc	4.80 a	2.26 b	47.47 a	0.28 ab
B. velezensis _306	33.33±1.15 bc	22.00	1.00 ab	0.54 a	53.94 a	4.20 a	1.84 a	45.01 a	0.25 ab
Control	26.00±1.73 a	/	0.80 ab	0.53 a	62.91 ab	5.10 a	2.23 b	52.11 a	0.24 ab

fw, fresh weight, dw, dry weight; DM dry matter; R/S, root to shoot dry weight ratio.

Gain%: the effect of bacterial treatments on plant height was assessed as relative percentage change in comparison to the control. Means in a column followed by the same letter are not significantly different according to Student–Newman–Keuls test (P≤ 0.05)
